# Low-dimensional magnetocaloric materials for energy-efficient magnetic refrigeration: does size matter?

**DOI:** 10.1080/14686996.2025.2546287

**Published:** 2025-08-13

**Authors:** Nguyen Thi My Duc, Hariharan Srikanth, Manh-Huong Phan

**Affiliations:** a Department of Physics, University of South Florida Tampa, FL, USA; b The University of Danang, University of Science and Education, Danang, Viet Nam; c Center for Materials Innovation and Technology, VinUniversity, Hanoi, Vietnam; d College of Engineering and Computer Science, VinUniversity, Hanoi, Vietnam

**Keywords:** Magnetocaloric materials, nanoparticles, thin films, ribbons, microwires, reduced dimensionality, magnetic refrigeration

## Abstract

The magnetocaloric effect (MCE) provides a promising foundation for the development of solid-state refrigeration technologies that could replace conventional gas compression-based cooling systems. Current research efforts primarily focus on identifying cost-effective magnetic materials that exhibit large MCEs under low magnetic fields across broad temperature ranges, thereby enhancing cooling efficiency. However, practical implementation of magnetic refrigeration requires more than bulk materials; real-world devices demand efficient thermal management and compact, scalable architectures, often achieved through laminate designs or miniaturized geometries. Magnetocaloric materials with reduced dimensionality, such as ribbons, thin films, microwires, and nanostructures, offer distinct advantages, including improved heat exchange, mechanical flexibility, and integration potential. Despite these benefits, a comprehensive understanding of how size, geometry, interfacial effects, strain, and surface phenomena influence the MCE remains limited. This review aims to address these knowledge gaps and provide guidance for the rational design and engineering of magnetocaloric materials tailored for high-performance, energy-efficient magnetic refrigeration systems.

## IMPACT STATEMENT

The magnetocaloric effect (MCE) offers a promising foundation for advancing solid-state refrigeration technologies, presenting a potential alternative to conventional gas compression-based cooling systems. Magnetocaloric materials with reduced dimensionality – such as ribbons, thin films, microwires, and nanostructures – present unique advantages, including enhanced heat transfer, mechanical flexibility, and ease of integration into compact devices. However, a comprehensive understanding of how factors such as size, geometry, interfacial interactions, strain, and surface effects influence the MCE remains incomplete. This review aims to bridge these knowledge gaps and provide insights to support the rational design and optimization of magnetocaloric materials for high-performance, energy-efficient magnetic refrigeration applications.

## GRAPHICAL ABSTRACT

**Figure:**
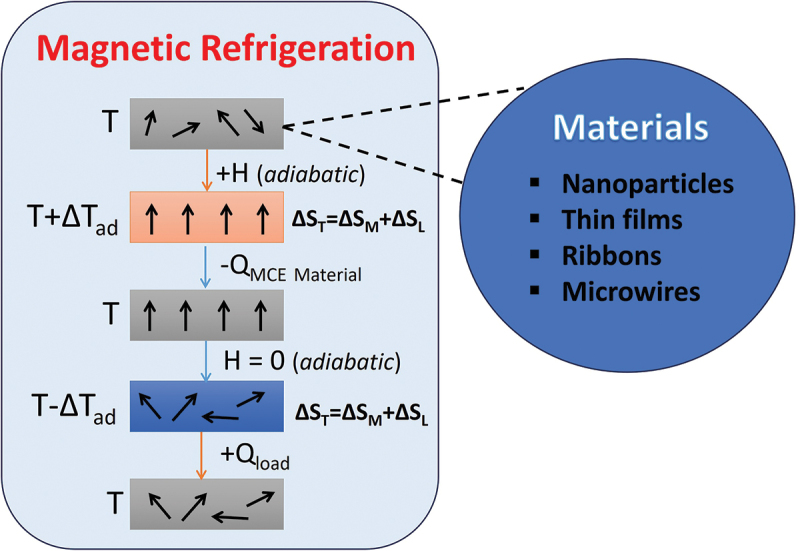

## 1. Introduction

Cooling technology is essential in modern life, supporting comfort, safety, technological performance, and environmental sustainability [1–3]. Contemporary cooling systems are increasingly focused on reducing energy consumption and minimizing the environmental impact of refrigerants to help mitigate climate change [1,2]. Vapor-compression cooling, based on gas compression and expansion, has long been the dominant method used in applications ranging from household air conditioners and supermarket refrigerators to refrigerated transport [1]. Its popularity stems from its cost-effectiveness and adaptability. However, these systems are energy-intensive, particularly in large-scale applications such as HVAC (Heating, Ventilation, and Air Conditioning) systems in commercial buildings or data centers, contributing significantly to electricity consumption, grid stress, and operational costs. Furthermore, the refrigerants used (e.g. CFCs, HCFCs) either deplete the ozone layer or possess a high global warming potential (GWP), raising serious environmental concerns. In addition, conventional cooling systems often require large, bulky components such as compressors, condensers, and evaporators, limiting their applicability in compact or mobile devices.

**Figure 1.**
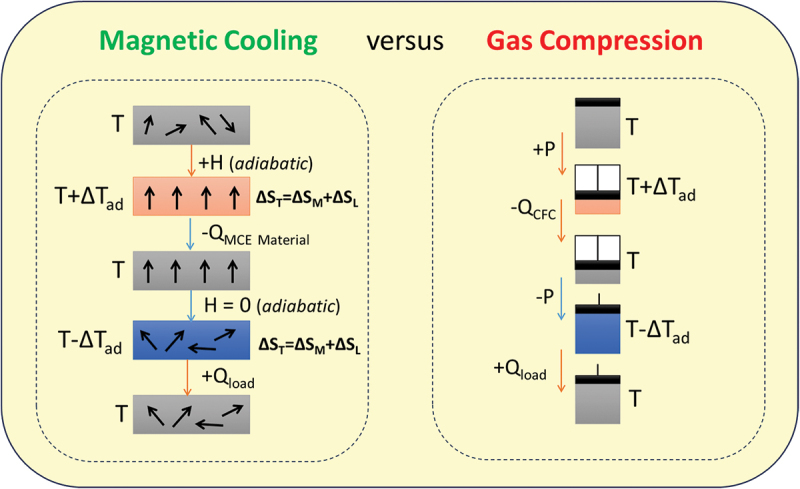
A full cooling cycle in magnetic refrigeration (left) differs fundamentally from that in conventional gas compression (right) techniques. In magnetic refrigeration, temperature changes are induced by cyclically magnetizing and demagnetizing a magnetocaloric material, which serves as the refrigerant. In contrast, gas compression refrigeration relies on compressing and expanding a gaseous refrigerant to produce pressure-induced temperature changes.

These limitations have driven significant research into alternative solid-state cooling technologies [1–5]. One of the most promising among them is magnetic refrigeration, which is based on the magnetocaloric effect (MCE) – a phenomenon in which a magnetic material heats up or cools down when subjected to a changing magnetic field [2,6]. When an external magnetic field is applied, the magnetic moments in the material align, reducing magnetic entropy and increasing the material’s temperature. Upon removal of the field, the moments become disordered again, increasing magnetic entropy and leading to cooling. This thermodynamic principle forms the foundation of magnetic refrigeration, offering a potential path toward environmentally friendly and energy-efficient cooling. Figure 1 illustrates the working principle of a magnetic cooling cycle and its advantages over traditional gas-compression systems.

MCE-based refrigeration systems offer several benefits, including the potential to operate without harmful refrigerants and with improved energy efficiency [6]. Since the efficiency of heat exchange in such systems depends on the magnetic entropy change (Δ*S*_*M*_) of the refrigerant material, materials exhibiting large Δ*S*_*M*_ are highly desirable [6–12]. This entropy change may be induced via magnetic or magneto-structural phase transitions, provided there is a significant change in magnetization between the two phases. Current research efforts focus on identifying materials that are both cost-effective and exhibit large Δ*S*_*M*_ under relatively low magnetic fields across a broad temperature range, resulting in a high refrigerant capacity (*RC*) [6–10], a key metric that quantifies the amount of heat transferred between the cold and hot reservoirs in an ideal refrigeration cycle [7].

Among the materials studied, gadolinium (Gd) is widely considered a benchmark for magnetic refrigeration near room temperature. It exhibits a large MCE and a second-order magnetic phase transition around 294 K [7,11]. Gd has been used in proof-of-concept devices demonstrating that magnetic refrigeration is a viable alternative with the potential for up to 30% energy savings compared to conventional methods [1,6]. However, Gd also suffers from several drawbacks that limit its scalability and commercial viability. As a rare-earth element, Gd is expensive and subject to supply chain vulnerabilities. Its moderate thermal conductivity may hinder heat transfer efficiency in packed-bed configurations. Additionally, it is prone to oxidation when exposed to air or moisture, which degrades its long-term performance [7].

In response to these challenges, a wide range of alternative magnetocaloric materials has been developed, including Gd₅(Ge₁-ₓSiₓ)₄ [13–15], La(Fe₁-ₓSiₓ)₁₃ [16], MnAs₁-ₓSbₓ [16], MnFeP₁-ₓAsₓ [17,18], Ni_50_Mn_50−x_Sn_x_ [19], and *R*₁-ₓ*T*ₓMnO₃ (*R* = La, Pr, Nd; *T* = Ca, Sr, Ba …) [8,20–22], among others [6,9,10]. While some of these materials show enhanced entropy changes or tunable transition temperatures [6–8], comparative studies have revealed that Gd still remains one of the most suitable candidates for sub-room temperature magnetic refrigeration due to its favorable balance of MCE performance, low hysteresis, and operational simplicity [9].

To realize magnetic cooling in practical devices, however, materials cannot always be used in bulk form [10,23–26]. Real-world systems require efficient thermal management and compact architectures, often implemented through laminate structures or miniaturized geometries. Magnetocaloric materials with reduced dimensionality, including ribbons, thin films, microwires, and nanostructures, offer significant advantages over their bulk counterparts in this regard [23–26]. For example, magnetocaloric ribbons, typically fabricated via rapid solidification (e.g. melt spinning), are thin (tens of microns) and exhibit high surface-area-to-volume ratios, which facilitate rapid heat exchange and efficient coupling with heat transfer fluids [23,24]. Their geometry also enables uniform exposure to magnetic fields and reduced demagnetization effects. Ribbons can be cut, stacked, or shaped to suit various device designs, offering excellent mechanical and processing flexibility. Similarly, thin films offer unique advantages for integration into micro- and nanoscale devices [25,27]. They can be deposited directly onto substrates for on-chip cooling in microelectronics, MEMS, or lab-on-chip platforms. Due to their low thermal mass and fast thermal response, thin films are especially promising for high-frequency cooling cycles. Theoretically, reducing the dimensions of magnetic refrigerants increases the cooling power by enabling higher operational frequencies [28,29]. Meanwhile, magnetocaloric wires, such as Gd alloy-based microwires [30–32], provide mechanical robustness and better control of fluid dynamics compared to spherical or irregular particles. Arrays of aligned wires have been shown to reduce viscous losses, improve temperature span, and enhance heat transfer performance [29]. The ability to assemble wire bundles into laminate configurations makes them suitable for compact and efficient magnetic cooling in MEMS (Micro-electro-mechanical systems) and NEMS (Nano-electro-mechanical systems) applications.

Despite these promising features, a comprehensive understanding of how geometrical constraints, interfaces, strain, and surface effects influence the magnetocaloric response is still lacking. While earlier reviews have focused largely on bulk magnetocaloric materials [6–9] or isolated studies of size effects [10,24–26], this review aims to provide a critical and comparative analysis of advanced magnetocaloric materials in reduced-dimensional forms (ribbons, thin films, nanoparticles, and microwires), highlighting the interplay between structural characteristics and magnetocaloric performance. This discussion is intended to guide the rational design and engineering of advanced magnetocaloric materials for next-generation, energy-efficient magnetic refrigeration technologies.

## 2. Criteria for selecting magnetocaloric materials

### 2.1. Magnetocaloric figures of merit

Selecting suitable magnetocaloric materials for use in magnetic refrigeration technologies involves balancing thermodynamic performance, physical properties, and practical considerations to ensure optimal efficiency, reliability, and scalability [6,7,23]. The primary requirement for a magnetic refrigerant is a large magnetocaloric effect (MCE) – quantified by the isothermal magnetic entropy change (Δ*S*_*M*_) and/or the adiabatic temperature change (Δ*T*_*ad*_) – under a relatively low magnetic field over a broad temperature range, resulting in a large refrigerant capacity (*RC*). These parameters determine the material’s capacity to transfer heat between thermal reservoirs during a magnetic refrigeration cycle (Figure 1).

The change in magnetic entropy (Δ*S*_M_(*T*,µ_0_*H*)) induced by varying the external magnetic field from *H* = 0 to *H* = *H₀* at a constant temperature, is commonly used to evaluate the MCE and is derived using Maxwell’s relation [7]:

(1) \Delta \it {S_M}(T,{H_0}) = {S_M}(T,{H_0}) - {S_M}(T,0) = {\mu _0} \mathop \int \limits_0^{H_0} {\left({{{\partial M\left({T,H} \right)} \over {\partial T}}} \right)} _H}dH

Here, *μ₀* is the vacuum permeability and (∂*M*/∂*T*)_*H*_ is the temperature derivative of magnetization at constant field. A material exhibiting a steep change in magnetization near its transition temperature will have a large (∂*M*/∂*T*)_*H*_, and consequently a large Δ*S*_*M*_ - a desirable feature in magnetic cooling materials.

Moreover, Δ*S*_*M*_ can be achieved from calorimetric measurements of the field dependence of the heat capacity and subsequent integration:

(2) \Delta \it S_{{M}}}\left({T,{H_0}} \right) = \mathop \int \limits_0^T {{C\left({T,H} \right) - C\left({T,0} \right)} \over T}dT

where *C*(*T*, *H*_0_) and *C*(*T*,0) are the values of the heat capacity measured in the field *H*_0_ and in zero magnetic field *H* = 0, respectively.

The adiabatic temperature change Δ*T*_*ad*_ in magnetocaloric materials can be formally described using the thermodynamic Maxwell relation as:

(3) $$\Delta T_{\mathrm{ad}}=-\overset{H}{\underset{0}{\int }}\frac{T}{C_{P}\left(T,H\right)}{\left(\frac{\mathrm{\partial M}\left(T,H\right)}{\mathrm{\partial T}}\right)}_{H}\mathrm{dH}$$

This fundamental equation indicates that Δ*T*_*ad*_ depends on both the heat capacity *C*_*p*_(*T,H*) and the temperature derivative of the magnetization ${\left(\frac{\mathrm{\partial M}}{\mathrm{\partial T}}\right)}_{H}$, integrated over the range of applied magnetic field. It provides a complete thermodynamic description of the adiabatic process during magnetization or demagnetization.

The adiabatic temperature change, Δ*T*_*ad*_, at a given temperature *T*_0_ can be estimated as:

(4) $$\Delta T_{\mathrm{ad}}(T_{0},H_{0})≅-\Delta S_{M}(T_{0},H_{0})\frac{T_{0}}{C(T_{0},H_{0})}$$

where *C*(*T,H*) is the specific heat under field. While a large Δ*S*_*M*_ contributes to a large Δ*T*_*ad*_, the specific heat can vary significantly between materials, meaning that high Δ*S*_*M*_ does not always guarantee high Δ*T*_*ad*_.

To more comprehensively assess the utility of a magnetocaloric material, its refrigerant capacity (*RC*) is typically considered as [7]:

(5) $$\mathrm{RC}=\int_{T_{\mathrm{hot}}}^{T_{\mathrm{cold}}}-\Delta S_{M}\left(T\right)dT,$$

Another related metric is the relative cooling power (*RCP*), defined as:

(6) $$\mathrm{RCP}=\;-\Delta {S_{M}}^{\max}\delta T_{\mathrm{FWHM}},$$

where δ*T*_FWHM_ = *T*_hot_ – *T*_cold_ is the full width at half maximum of the Δ*S*_*M*_(*T*) peak. Both *RC* and *RCP* provide insight into the effectiveness of a material across a practical temperature span. It is important to note that the *RC* does not correspond to the mechanical work performed during a thermodynamic cycle. Instead, it serves as a thermodynamic performance metric that quantifies the total heat transferred between the cold and hot reservoirs during a magnetization – demagnetization cycle of a magnetocaloric material. Description (Eq. 5) refers to modern active magnetic refrigeration (AMR) cycles, which often utilize stacked magnetic refrigerants arranged in parallel. In these systems, magnetocaloric materials are layered so that each operates optimally within a specific segment of the overall temperature span. Heat transfer fluid flows in parallel through the stack, facilitating efficient thermal exchange across the entire bed. This configuration enhances the regenerative heat transfer process, increasing both the cooling span and the overall efficiency of the system.

**Figure 2.**
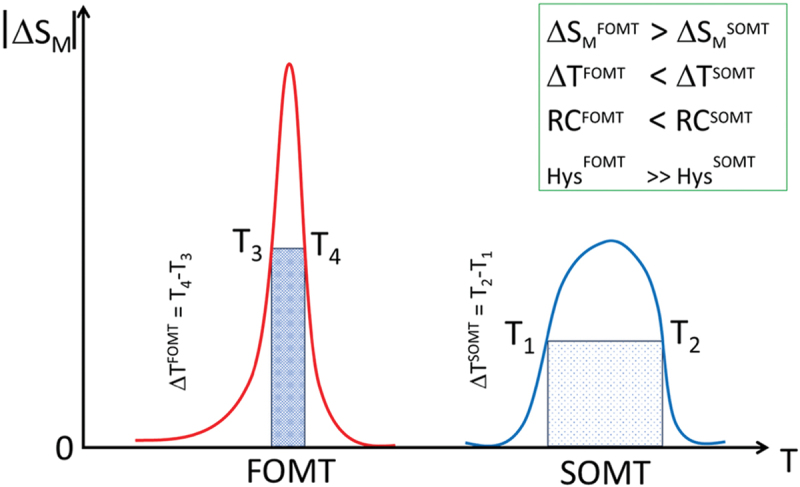
Schematic illustration of the magnetic entropy change (|Δ*S*_*M*_*|*) as a function of temperature for materials exhibiting first-order magnetic transitions (FOMT) and second-order magnetic transitions (SOMT). FOMT materials typically display a larger Δ*S*_*M*_ confined to a narrow temperature range (Δ*T*^*FOMT*^ = *T*_4_-*T*_3_), often accompanied by significant magnetic and thermal hysteresis (*Hys*^*FOMT*^). in contrast, SOMT materials exhibit a smaller Δ*S*_*M*_ over a broader temperature span (Δ*T*^*SOMT*^ = *T*_2_-*T*_1_) with minimal hysteresis (*Hys*^*SOMT*^), which can even result in a higher *RC*.

Magnetocaloric materials are broadly classified by their magnetic phase transitions. *First-order magnetic transition (FOMT)* materials exhibit an abrupt change in magnetization, often coupled with structural or volume changes. These materials typically show a large Δ*S*_*M*_ but within a narrow temperature range and often with magnetic or thermal hysteresis, which can reduce efficiency and reversibility [12,19]. *Second-order magnetic transition (SOMT)* materials, on the other hand, undergo a continuous magnetization change near the Curie temperature (*T*_*C*_), without structural changes. Although Δ*S*_*M*_ is lower than in FOMT materials, SOMT materials such as Gd offer broader operating ranges, minimal hysteresis, and superior thermal and mechanical stability – favorable traits for cyclic refrigeration [6,7,12]. Figure 2 illustrates a general trend of the temperature dependence of magnetic entropy change for both FOMT and SOMT materials. Because hysteresis leads to energy loss and heating, materials with soft magnetic behavior and negligible hysteresis are preferable [23]. SOMT materials are particularly suitable for high-frequency and long-term refrigeration applications.

Additional practical criteria for selecting magnetocaloric materials include: (i) *high thermal conductivity*, to enable rapid and efficient heat exchange; (ii) *low electrical conductivity*, to reduce eddy current losses during dynamic magnetic field cycles, thus preserving the cooling efficiency and enabling more efficient, compact, and faster-operating refrigeration; (iii) *resistance to oxidation and corrosion*, to ensure durability under repeated magnetic and thermal cycling; (iv) *mechanical robustness*, essential for long-term stability and reliable device integration; (v) *environmental safety*, requiring materials to be non-toxic and free from hazardous elements; (vi) *cost-effectiveness and availability*, as abundant and low-cost materials are more suitable for large-scale commercialization; and (vii) *formability and processability*, allowing adaptation to diverse device architectures such as thin films, ribbons, and microwires.

### 2.2. Notable advantages of reduced dimensionality

While bulk materials often suffer from limited heat exchange surface area [6,7], reduced-dimensional forms, such as nanoparticles, thin films, ribbons, or microwires, can offer enhanced heat transfer, flexibility, and integration into compact systems [10,23–30]. Nanoparticles, for example, are particularly attractive for cryogenic and localized cooling due to their scalability and adaptability, and their inherent entropy broadening can contribute to an enhanced RC. Thin films, on the other hand, exhibit strong potential for on-chip and microscale cooling applications, and can be integrated with other physical effects such as thermoelectricity. Ribbons provide high surface area, fast thermal response, and moderate mechanical flexibility, making them promising for rapid heat exchange environments. Microwires further combine a high surface-to-volume ratio with excellent mechanical flexibility, enabling effective wrapping around heat sources and fast thermal coupling with their surroundings. Kuz’min theoretically demonstrated that magnetic refrigerators have an upper operational frequency limit of approximately 200 Hz [28]. This maximum frequency is governed by the minimum delay between switching off the magnetic field and the subsequent transfer of the induced temperature change to the heat exchanger. The key limitation on operational frequency arises from a trade-off between thermal conductivity and viscous friction. Mechanical instability, often caused by flow maldistribution, can also reduce system throughput significantly. Unlike bulk materials, magnetocaloric materials in wire form, especially when arranged in wire bundles within the magnetic bed, are predicted to offer enhanced mechanical stability and lower porosity, making them more suitable for high-frequency operation [28]. D. Vuarnoz and T. Kawanami conducted an extensive analysis of pressure drop, refrigeration capacity, coefficient of performance (COP), and exergy efficiency in a reciprocating active magnetic regenerator (AMR) composed of gadolinium wires [29]. Their findings indicate that smaller wire diameters significantly improve both cooling capacity and COP. This improvement is attributed to the increased heat transfer surface area and reduced interstitial space between wires, which together enhance the convective heat transfer coefficient. For a given wire diameter, an AMR utilizing a wire stack outperforms one with a particle bed, delivering superior overall performance [29]. Nonetheless, it should be noted that the increased surface area in reduced-dimensionality materials can also lead to higher friction, which may offset the benefits of enhanced heat exchange. For practical cooling applications, the trade-off between heat transfer efficiency and pressure drop must be carefully considered, not only in terms of optimizing the size and shape of the magnetocaloric materials, but also with respect to the choice of matrix materials in which magnetocaloric components, such as magnetic nanoparticles or microwires, are embedded. These aspects will be further explored in the next section, where the role of reduced dimensionality and associated effects (e.g. strain, surface/interface phenomena) on the MCE response will be critically examined.

## 3. Magnetocaloric materials: reduced dimensionality effects

The MCE is influenced differently by reduced dimensionality across various types of magnetic ordering – ferromagnetic, antiferromagnetic, and ferrimagnetic. These effects can differ significantly when comparing bulk materials to low-dimensional forms such as nanoparticles, thin films, ribbons, and microwires. We note herein that the term ‘low-dimensional materials’, as used in this paper, broadly refers to materials with reduced dimensionality, such as thin films, nanoparticles, ribbons, and microwires. It is not intended to be limited solely to atomically thin materials or systems exhibiting dimensional confinement at the atomic scale. Additionally, phase coexistence has been shown to markedly impact the MCE in bulk systems and may interact with reduced dimensionality effects in complex ways [31,33–35]. In this section, we examine how these factors influence the MCE response in each form of reduced-dimensional magnetic material, including nanoparticles, thin films, ribbons, and microwires.

### 3.1. Nanoparticles

Finite size and surface effects are critical factors that significantly influence the magnetic behavior of nanoparticles compared to their bulk counterparts. Finite size effects stem from the limited number of atoms and reduced dimensions of nanoparticles, typically below 100 nm [36–38]. As particle size decreases, thermal fluctuations become more pronounced, often disrupting magnetic ordering and suppressing long-range magnetic interactions. Consequently, magnetic transition temperatures such as the Curie temperature (*T*_*C*_) or Néel temperature (*T*_*N*_) tend to decrease due to reduced coordination of magnetic atoms and the enhanced surface-to-volume ratio [36]. At sufficiently small sizes, nanoparticles transition to single-domain states, altering coercivity and magnetization reversal behavior, and often giving rise to superparamagnetism [39].

The high surface-to-volume ratio also means that a large fraction of atoms reside at or near the surface, where they experience altered chemical and magnetic environments. These surface atoms have fewer nearest neighbors, leading to broken magnetic exchange bonds and spin frustration or canting, which reduces overall magnetization [36,40,41]. The surface’s reduced symmetry enhances magnetic anisotropy, often dominating over bulk contributions and affecting magnetization dynamics. In certain ferro/ferrimagnetic systems, the surface may become magnetically inactive (a so-called ‘dead layer’) or develop distinct magnetic properties, forming core-shell structures—e.g. a ferro/ferrimagnetic core with a spin-glass-like shell [40,42,43]. Such surface-induced spin disorder and enhanced anisotropy can increase coercivity in single-domain nanoparticles.

**Figure 3.**
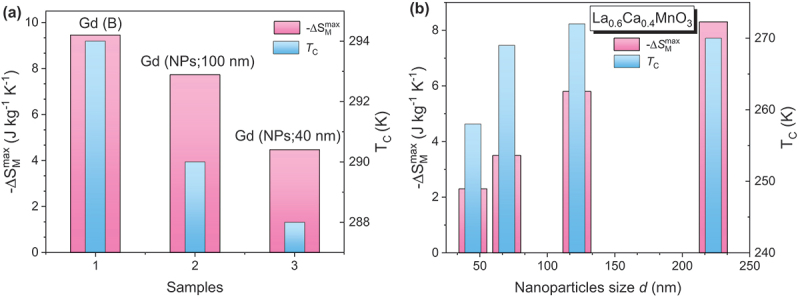
Maximum magnetic entropy change (−Δ*S*_*M*_^*max*^) and Curie temperature (*T*_*C*_) for bulk Gd and Gd nanoparticles with grain sizes of 100 nm and 15 nm under a magnetic field change of 5 T; (b) −Δ*S*_*M*_^*max*^ and *T*_*C*_ as functions of nanoparticle grain size under a magnetic field change of 5 T.

As a result of these size and surface effects, the magnetocaloric response in nanoparticles often deviates markedly from that in bulk materials (see Table 1). For ferromagnets, reducing particle size generally leads to decreases in *T*_*C*_, saturation magnetization (*M*_*S*_), and Δ*S*_*M*_ [44–47]. For instance, in Gd, -Δ*S*_*M*_^max^ and *RCP* decrease from 9.45 J/kg·K and 690 J/K (bulk) to 7.73 J/kg·K and 234 J/K (100 nm), and further to 4.47 J/kg·K and 140 J/K (15 nm), with corresponding decreases in *T*_*C*_ from 294 K to 290 K and 288 K (see Figure 3(a)) [44,45]. Interestingly, ferromagnetic nanoparticles often exhibit a broader distribution of Δ*S*_*M*_(*T*) compared to their bulk forms, which can sometimes enhance *RCP* despite a lower peak Δ*S*_*M*_. For example, Gd_5_Si_4_ nanoparticles produced via ball milling show a reduced peak Δ*S*_*M*_ and a shift to lower temperatures, but the broader Δ*S*_*M*_ (*T*) profile results in a 75% *RCP* increase [46].

**Table 1.** Maximum entropy change, $\left|\Delta S_{M}^{\max}\right|$, Curie temperature, *T*_C_, refrigerant capacity (*RC*), and relative cooling power (*RCP*) for the nanoparticle samples.

Samples	Size (nm)	*T*_C_ (K)	µ_0_∆*H* (T)	$\left|\Delta S_{M}^{\max}\right|$ (J/kg K)	*RC* (J/kg)	*RCP* (J/kg)	Ref.
***Gadolinium and its alloys***							
Gd (*B*)	*B*	294	1 2 5	2.8 5.07 9.45	–	63.4 187 690	[44]
Gd (*NPs; C*)	100 15	290 288	5 5	7.73 4.47	–	234 140	[45]
Gd_5_Si_2_Ge_2_ (*NPs; C*)	85	225	2	0.45	–	–	[65]
GdNi_5_ (*NPs; C*)	15	31	5	13.5	–	–	[66]
Gd_5_Si_4_ (*B*) *NPs; milled 2 h* *NPs; milled 3 h*	*B* 420 360	340 320 320	3 3 3	~6.5 ~3 < 3	–	~200 ~340 ~340	[46]
***Oxides***							
LaMnO_3_ (*B*)	*B*	124	5	2.69	170	250	[62]
LaMnO_3_ (*NPs; C*) Annealed at 1000°C	200	135	5	2.67	282	355	[62]
LaMnO_3_ (*NPs; C*) Annealed at 800°C	40	150	5	2.4	284	369	[62]
La_0.125_Ca_0.875_MnO_3_ (*B*)	*B*	123	7	6.3	–	63.1	[67]
La_0.125_Ca_0.875_MnO_3_ (*NPs; C*)	70	113	7	1.32	–	22.8	[67]
La_0.4_Ca_0.6_MnO_3_ (*NPs; C*)	130	260	5	2.81	240.7	–	[51]
La_0.4_Ca_0.6_MnO_3_ (*NPs; C*)	50	100	5	0.33	46.6	–	[51]
La_0.4_Ca_0.6_MnO_3_ (*NPs; C*)	25	80	5	0.13	11	–	[51]
La_0.4_Ca_0.6_MnO_3_ (*NPs; A*)	10	45	5	0.46	8.1		[51]
La_0.5_Ca_0.5_MnO_3_ (*NPs; C*)	8.3	245	2	0.75	–	93	[68]
La_0.6_Ca_0.4_MnO_3_ (*B*)	*B*	264	5	5.5	–	139	[69]
La_0.6_Ca_0.4_MnO_3_ (*NPs; C*)	223	270	5	8.3	–	508	[47]
La_0.6_Ca_0.4_MnO_3_ (*NPs; C*)	122	272	5	5.8	–	374	[47]
La_0.6_Ca_0.4_MnO_3_ (*NPs; C*)	70	269	5	3.5	–	251	[47]
La_0.6_Ca_0.4_MnO_3_ (*NPs; C*)	45	258	5	2.3	–	228	[47]
La_0.6_Ca_0.4_MnO_3_ (*NT; C*)	23	280	5	0.3	–	40	[47]
La_0.6_Ca_0.4_MnO_3_ *NPs; C; sol-gen*	45	258	1	0.6	–	50	[47]
La_0.67_Ca_0.33_MnO_3_ (*B*)	*B*	258	1	5	56.2	55	[70] (*)
La_0.67_Ca_0.33_MnO_3_ (*NPs; C*)	60	250	1	1.75	33.5	–	[70]
La_0.67_Ca_0.33_MnO_3_ (*NPs; C*)	20	260	1	0.2	–	25.6	[62]
La_0.7_Ca_0.3_MnO_3_ (*B*)	*B*	264	5	7.8	187	~280	[52] (*)
La_0.7_Ca_0.3_MnO_3_ (*NPs; C*)	33	260	5	4.9	146	~170	[52]
La_0.7_Ca_0.3_MnO_3_ (*NPs; C*)	15	241	5	2.4	162.5	~180	[52]
La_0.7_Ca_0.3_MnO_3_ (*F; C*)	150	235	5	2.75	200	~260	[52]
La_0.7_Ca_0.3_MnO_3_ (*B*)	*B*	235	4.5	6.99	243.1	–	[71]
La_0.7_Ca_0.3_MnO_3_ (*NPs; C*)	160	270	4.5	5.02	218.4	–	[71]
La_0.7_Ca_0.3_MnO_3_ (*NPs; C*)	65	266	1.5	1.2	–	44	[72]
La_0.8_Ca_0.2_MnO_3_ (*NPs; C*)	17	234	4.5	0.6	–	150	[53]
La_0.8_Ca_0.2_MnO_3_ (*NPs; C*)	28	214	4.5	4.5	–	350	[53]
La_0.8_Ca_0.2_MnO_3_ (*NPs; C*)	43	236	4.5	8.6	–	200	[53]
La_0.67_Sr_0.33_MnO_3_ (*B*)	*B*	377	2	2.02	–	101	[73]
La_0.67_Sr_0.33_MnO_3_ (*B*)	*B*	370	1	1.5	–	42	[74]
La_0.67_Sr_0.33_MnO_3_ (*NPs; C*)	80	354	2 5	1.15 2.49	–	88 225	[44]
La_0.67_Sr_0.33_MnO_3_ (*NPs; C*)	85	369	1.5	1.74	–	52	[50]
La_0.67_Sr_0.33_MnO_3_ (*NPs; C*)	51	367	1.5	1.3	–	48	[50]
La_0.67_Sr_0.33_MnO_3_ (*NPs; C*)	32	362	1.5	0.32	–	20	[50]
La_0.67_Sr_0.33_MnO_3_ (*F*)	2400	348	5	1.69	–	211	[75]
La_0.67_Sr_0.33_MnO_3_ (*B*)	*B*	370 370	2 5	2.68 5.15	–	85 252	[76]
La_0.8_Sr_0.2_MnO_3_ (*B*)	*B*	301	2	2.2	–	35	[77]
La_0.8_Sr_0.2_MnO_3_ (*NPs; C*)	23	295	2	0.5	–	32	[77]
Pr_0.67_Sr_0.33_MnO_3_ (*NPs; C*)	80	258	2 5	0.82 1.94	–	99 265	[44]
Pr_0.7_Sr_0.3_MnO_3_ (*NPs; C*)	35	235	5	6.3	–	385	[78]
Pr_0.67_Sr_0.33_MnO_3_ (*B*)	*B*	281	5	7.8	–	195	[79]
Pr_0.67_Sr_0.33_MnO_3_ (*B*)	*B*	260	1	1.75	–	49	[80]
Nd_0.67_Sr_0.33_MnO_3_ (*NPs; C*)	80	206	2 5	0.35 0.93	–	87 246	[44]
Nd_0.63_Sr_0.37_MnO_3_ (*SC*)	*B*	300	5	8.25	–	511	[21]
Pr_0.65_(Ca_0.7_Sr_0.3_)_0.35_MnO_3_ (*B*)	*B*	215	7	7.8	273	312	[81]
Pr_0.65_(Ca_0.7_Sr_0.3_)_0.35_MnO_3_ (*NPs; C*)	67	225	5	6.0	180	142	[82]
La_0.35_Pr_0.275_Ca_0.375_MnO_3_ (*B*)	*B*	75	5	4.5	34.64	–	[63]
La_0.35_Pr_0.275_Ca_0.375_MnO_3_ (*NPs; C*)	50	215	1 5	2.94 6.2	37.2 225.6	–	[63]
La_0.215_Pr_0.41_Ca_0.375_MnO_3_ (*B*)	*B*	210	5	5.3	143.1	–	[63]
La_0.7_Ca_0.3_Mn_0.9_Ni_0.1_O_3_ (*NPs; C; BM*)	15	145	1.5	0.95	–	–	[83]
La_0.7_Ca_0.2_Sr_0.1_MnO_3_ (*NPs; C; HEBM*)	150–300	308	1.8	4.11	–	61.12	[84]
DyCrTiO_5_ (*NPs; C; exchange bias*)	37	153 (*T*_N_)	3	10.9 @10 K	–	~76.3	[60]
Tb_2_O_3_ (*NPs; C*)	51	8 (*T*_N_)	6	6.6	53.9	–	[61]
Dy_2_O_3_ (*NPs; C*)	68	4 (*T*_N_)	6	18.2	46.5	–	[61]
Gd_2_O_3_ (*NPs; C*)	44	3.5 (*T*_N_)	6	23.2	–	–	[61]
Ho_2_O_3_ (*NPs; C*)	56	2 (*T*_N_)	6	31.9	–	–	[61]
GdVO_4_ (*NPs; C*)	30	2.5	7	33	–	–	[85]
GdVO_4_ (*NPs; C*)	300	2.5	7	45	–	–	[85]
GdVO_4_ (*B*)	2500	2.5	7	43	–	–	[85]
GdVO_4_ (*B*)	5000	2.5	7	30	–	–	[85]
Gd_3_Fe_5_O_12_ (*B*)	*B*	35 35	1 3	0.78 2.45	–	-288	[58]
Gd_3_Fe_5_O_12_ (*NPs; C*)	50	25 25	1 3	0.31 1.49	–	–	[58]
Gd_3_Fe_5_O_12_ (*NPs; C*)	35	5 5	1 3	0.67 3.47	–	–	[58]
***Austenitic alloys***							
γ-FeNiMn *NPs; C; BM 10 h*	17	340	1	0.41	–	78	[86]
(Fe_70_Ni_30_)_99_Cr_1_ *NPs; C; BM*	12	398 398	1 5	0.38 1.58	–	82 548	[64]
(Fe_70_Ni_30_)_97_Cr_3_ *NPs; C; BM*	10	323 323	1 5	0.27 1.49	–	59 436	[64]
(Fe_70_Ni_30_)_95_Cr_5_ *NPs; C; BM*	13	258 258	1 5	0.37 1.45	–	77 406	[64]
(Fe_70_Ni_30_)_94_Cr_6_ *NPs; C; BM*	12	245 245	1 5	0.29 1.22	–	62 366	[64]
(Fe_70_Ni_30_)_93_Cr_7_ *NPs; C; BM*	11	215 215	1 5	0.28 1.11	–	47 306	[64]
***Others***							
Co *NPs; C* Co_core_Ag_shell_ *NPs; C*	50 40 *core* 28 *shell*	15 15 15 20 20 20	1 2 3 1 2 3	1.00 1.75 2.35 0.82 1.16 2.28	~10.46 ~19.01 ~26.85 ~5.12 ~10.65 ~15.78	~10.92 ~20.40 ~28.20 ~5.4 ~11.4 ~16.8	[48]
Ni_100-x_Cr_x_ (*NPs; C*) *x* = 0 *x* = 5 *x* = 10 *x* = 15	4.7 5.1 5.6 5.9	614 550 349 147	0.1 0.1 0.1 0.1	0.15 0.10 0.05 0.03	–	23.30 33.45 21.97 13.17	[87]
Pr_2_Fe_17_ (*NPs; C; BM 40 h*)	11	285	1.5	0.6		60	[88]
Nd_2_Fe_17_ (*NPs; C; BM 40 h*)	11	337	1.5	1	–	118	[88]
Co_2_FeAl (*NPs; C*)	16	1261	1.4	15	–	89	[89]
Ni_50_Mn_34_In_16_ (*NPs; C*)	150	226–241	6	2	–	150	[90]
MnPS_3_ *TM thiophosphate*	20–50	2.85 2.85	3 9	6.8 12.8	–	–	[91]
MnFeP_0.45_Si_0.55_ *B, HEBM 0 h* *NPs, HEBM 26 h* *NPs, HEBM 26 h, 600* ^*0*^*C*	27000 31.6 31.6	392 390 382.1	1 1 1 2	2.8 0.8 1.2 2.4	–	--29 77	[92]
Fe_47.5_Ni_37.5_Mn_15_ (*NPs; C*)	7.5	327	5	1.3	–	297.68	[93]
γ-(Fe_70_Ni_30_)_89_Zr_7_B_4_ (*NPs; C; BM*)	20	353	1.5	0.7	–	65	[94]

A: Amorphous; C: Crystalline; NPs: Nanoparticles; F: Films; B: Bulk; P: Powder; SC: Single crystal; NT: Nanotubes. HEBM: High energy ball milling. (*) represents FOMT materials.

In Co nanoparticles (~50 nm), Poddar *et al*. reported a surface spin order – disorder transition at low temperatures associated with a significant MCE, alongside a superparamagnetic transition with a smaller magnetic entropy change at higher temperatures [48]. Surface spins were further manipulated by Ag shell coatings, forming Co/Ag core-shell structures that altered the MCE response. This illustrates how surface anisotropy and exchange coupling at the core-shell interface can be engineered to tailor magnetocaloric properties for magnetic refrigeration applications. Size reduction also enhances low-temperature MCE in Eu_8_Ga_16_Ge_30_ clathrate nanocrystals prepared by ball milling [49]. For 15 nm particles, -Δ*S*_*M*_^max^ reaches ~10 J/kg·K at 5 K under a 5 T field, attributed to modified interactions between Eu^2 +^ ions at distinct crystallographic sites.

Among magnetocaloric nanosystems, manganese oxides have been extensively studied due to their tunable magnetic and magnetocaloric properties via dopant concentration [47,50–53]. Similar to Gd, a trend of decreasing Δ*S*_*M*_, *RC* (*RCP*), and *T*_*C*_ with decreasing particle size has been observed in La₀.₆Ca₀.₄MnO₃ [47] and La₀.₇Ca₀.₃MnO₃ [52]. For the latter, -Δ*S*_*M*_^max^ and *RC* reduce from 7.7 J/kg·K and ~270 J/K (bulk) to 4.9 J/kg·K and ~200 J/K (35 nm), and 2.4 J/kg·K and ~150 J/K (15 nm), while *T*_*C*_ drops from 264 K to 260 K and 241 K (see Figure 3(b)). The decrease in Δ*S*_*M*_ correlates with reduced *M*_*S*_, often attributed to surface spin disorder. Lampen *et al*. estimated a 1.2 nm dead magnetic layer in 15 nm La₀.₇Ca₀.₃MnO₃ nanoparticles based on geometric arguments [52]. Table 1 shows variations in Δ*S*_*M*_ and *RC* (*RCP*) for samples with nominally identical compositions, likely due to oxygen off-stoichiometry – an important parameter that should be accurately reported in future studies for proper comparison.

In ferrimagnets such as Fe₃O₄, NiFe₂O₄, and CoFe₂O₄, nanosizing typically induces superparamagnetism, with thermal energy overcoming anisotropy barriers, resulting in rapid magnetic moment fluctuations and surface spin freezing at low temperatures [54–57]. CoFe₂O₄ nanoparticles exhibit a small Δ*S*_*M*_ around the blocking temperature, while a larger entropy change occurs below the spin-freezing point [54]. However, the magnitude of Δ*S*_*M*_ around the blocking temperature is often insufficient for practical refrigeration. This trend is commonly observed in a wide range of ferrite nanoparticle systems.

Some ferrimagnetic nanoparticle systems benefit from surface spin freezing, which increases *M* and Δ*S*_*M*_ under high magnetic fields. In Gd₃Fe₅O₁₂ (gadolinium iron garnet), Phan *et al*. observed that -Δ*S*_*M*_^max^ increased from 2.45 J/kg·K at 35 K (bulk) to 4.47 J/kg·K at 5 K for 35 nm nanoparticles under a 3 T field [58]. The enhancement is attributed to both the intrinsic magnetic frustration of the Gd sublattice and surface spin disorder. Applying sufficiently high magnetic fields effectively suppresses these disordered and frustrated spins, leading to a significant change in magnetization and, consequently, a large magnetic entropy change.

In antiferromagnetic nanoparticles, size reduction weakens AFM couplings and can induce weak ferromagnetism at the surface [59–61]. Under strong magnetic fields, AFM order may be suppressed in favor of FM alignment, increasing magnetization and magnetic entropy change. Notable examples include Tb₂O₃, Dy₂O₃, Gd₂O₃, and Ho₂O₃ nanoparticles [61]. For Ho₂O₃, Boutahar *et al*. reported large Δ*S*_*M*_ and *RCP* values of 31.9 J/kg·K and 180 J/K, respectively, near *T*_*N*_ ~2 K under a 5 T field [61].

In mixed-phase systems containing coexisting FM and AFM regions, nanosizing has been reported to enhance both Δ*S*_*M*_ and *RC* (*RCP*) [62,63]. Unlike single-phase FM systems where *T*_*C*_, Δ*S*_*M*_, and *RCP* tend to decrease with reducing particle size, Phan *et al*. observed the opposite trend in mixed-phase La₀.₃₅Pr₀.₂₇₅Ca₀.₃₇₅MnO₃ nanoparticles (~50 nm) [63]. Here, nanosizing suppressed the AFM state and promoted FM ordering, enabling a large Δ*S*_*M*_ and *RC* (*RCP*) at relatively low magnetic fields (~2 T). For a 5 T field, *RC* increased from ~61 J/kg (bulk) to ~225 J/kg (nanoparticles), while thermal and magnetic hysteresis losses, due to the FOMT characteristics of the material, were also significantly reduced. The magnetocaloric properties in such systems can be further tuned by adjusting the FM/AFM phase volume fractions, presenting a promising strategy for developing efficient nanostructured magnetocaloric materials.

The MCE has also been investigated in ball-milled nanoparticles of austenitic alloys, such as (Fe_70_Ni_30_)_99-x_Cr_1+x_ [64]. The addition of Cr significantly lowers the *T*_*C*_, from 398 K at *x* = 0 to 215 K at *x* = 6. This Cr substitution slightly reduces the maximum magnetic entropy change (-Δ*S*_*M*_^max^), from 1.58 J/kg·K to 1.11 J/kg·K under a magnetic field change of 5 T. Other nanoparticle systems studied for their MCE properties [65–94] are also summarized in Table 1.

### 3.2. Thin films

Similar to magnetic nanoparticles, finite size and surface effects in magnetic thin films significantly influence their magnetic and magnetocaloric properties compared to bulk materials [25,33,95,96]. These effects arise from the reduced dimensionality (typically nanometer-scale thickness) and the high surface-to-volume ratio inherent to thin-film systems. Finite size effects become prominent when film thickness approaches characteristic magnetic length scales, such as the exchange length or domain wall width [25]. Reduced atomic coordination along the thickness direction and enhanced thermal fluctuations in two-dimensional (2D) systems generally lead to a decrease in the *T*_*C*_ or *T*_*N*_ temperature with decreasing film thickness [95–152].

Magnetic properties in thin films are highly sensitive to parameters such as thickness, substrate, deposition method, annealing conditions, and oxygen stoichiometry [25,97,101–106,127,136–139]. For ferromagnetic films, reductions in thickness typically lead to suppression of *T*_*C*_, *M*_*S*_, and Δ*S*_*M*_. In moderately thick films ( >100 nm), these changes are often attributed to disorder from strain relaxation, which introduces defects like dislocations, vacancies, and grain boundaries [25,52,104,106]. However, in ultrathin, coherently strained films, distinguishing the effects of strain on the magnetic properties from intrinsic finite size, surface and interface phenomena remains a topic of debate.

**Figure 4.**
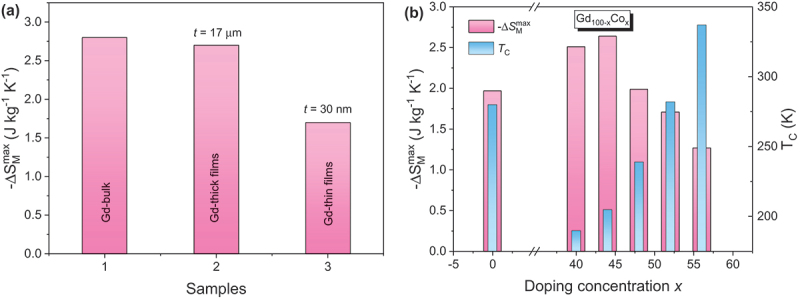
(a) Temperature dependence of the magnetic entropy change (−Δ*S*_*M*_) for bulk, thick films, and thin films Gd, showing a clear enhancement of −Δ*S*_*M*_^*max*^ with increasing film thickness under a field change of 1 T; (b) maximum magnetic entropy change (−Δ*S*_*M*_^*max*^) and Curie temperature (*T*_*C*_) as functions of Co-doping concentration for Gd_100-x_Co_x_ (*x* = 0–56) thin films under a field change of 2 T.

For instance, in Gd thin films, high Δ*S*_*M*_ values observed in the bulk [25] are maintained in thick films [97] but diminish significantly in thinner layers [25]. Under a 1 T field, -Δ*S*_*M*_^max^ drops from 2.8 J/kg·K in bulk to 2.7 J/kg·K at 17 μm thickness and to 1.7 J/kg·K at 30 nm (Figure 4(a)), while *T*_*C*_ remains nearly unchanged (~292–294 K). Interestingly, *RCP* increases from 63 J/kg (bulk) to 140 J/kg (17 μm) and 110 J/kg (30 nm), due to broadening of the Δ*S*_*M*_(*T*) – a consequence of dimensionality, surface, and interface effects on the magnetic phase transition. Thin films inherently include two key interfaces – film/substrate and film/capping layer – where atomic coordination is broken, leading to spin canting or non-collinear spin structures that reduce net magnetization. Polarized neutron reflectometry, for example, has revealed suppressed magnetic moments at Gd/W interfaces in Gd(30 nm)/W(5 nm) multilayers, contributing to reduced Δ*S*_*M*_ compared to bulk Gd [98].

In alloyed thin films like Gd_100–x_Co_x_ (100 nm thick), varying the Gd/Co ratio significantly affects both *T*_*C*_ and Δ*S*_*M*_ [99]. While increasing Co concentration generally raises *T*_*C*_ (except at *x* = 0), -Δ*S*_*M*_^max^ and *RCP* exhibit nonlinear dependencies, peaking for Gd_56_Co_44_ (see Figure 4(b)). Similarly, in Gd_x_(Fe_10_Co_90_)_100–x_ films (90 nm thick), increasing Gd content shifts *T*_*C*_ from 436 K to 558 K, with the -Δ*S*_*M*_^max^ observed at *x* = 50.

**Figure 5.**
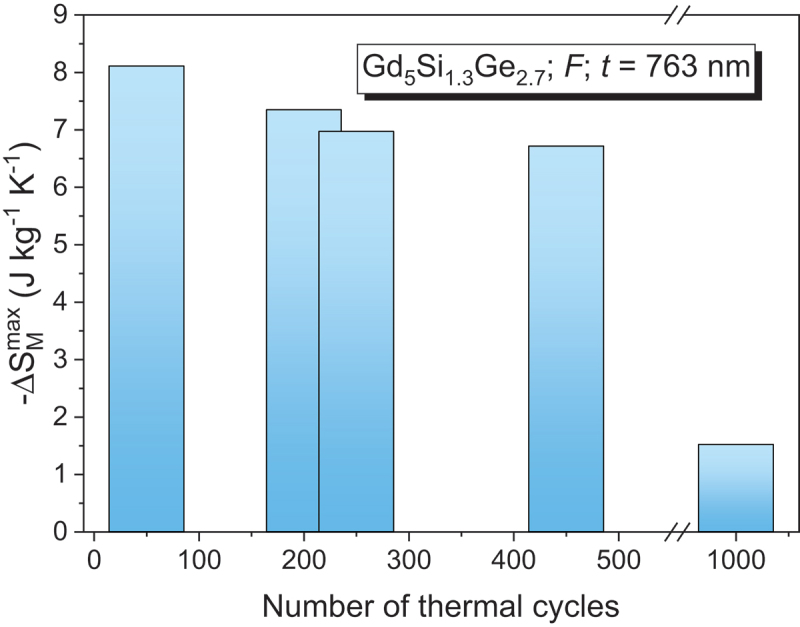
Maximum magnetic entropy change (−Δ*S*_*M*_^max^) as a function of the number of thermal cycles, showing a clear decrease in −Δ*S*_*M*_^max^ with increasing thermal cycling for Gd_5_Si_1.3_Ge_2.7_ thin films under a field change of 5 T.

In contrast to the giant Δ*S*_*M*_ of 18.4 J/kg·K reported for bulk Gd_5_Si_2_Ge_2_ (*T*_*C*_ = 276 K) [45], its film analog, Gd_5_Si_1.3_Ge_2.7_, shows a lower -Δ*S*_*M*_^max^ (~8.8 J/kg·K at 194 K under 5 T) [100]. This reflects both compositional changes and size effects. Notably, thermal cycling in these films leads to degradation of magnetocaloric performance: after 1000 cycles, -Δ*S*_*M*_^max^ drops from 8.1 to 1.52 J/kg·K (see Figure 5), underscoring a key limitation of FOMT materials in magnetic refrigeration technology [101].

In Heusler alloy films (e.g. Ni_53.4_Mn_33.2_Sn_13.4_ and Ni_53.2_Mn_29.2_Co_7.0_Sn_10.6_), decreasing film thickness from 1000 to 360 nm lowers -Δ*S*_*M*_^max^ and *T*_*C*_, though high *T*_*C*_ values are retained [102]. While -Δ*S*_*M*_^max^ values are modest (≤1.2 J/kg·K), these films are still relevant due to their tunability and potential multicaloric applications. Some Heusler alloy films of other compositions exhibit larger -Δ*S*_*M*_^max^ values but relatively small *RC* values (see Table 2). It is also noteworthy from Table 2 that while some Heusler alloy films exhibit large magnetic entropy changes near their FOMT temperatures, these changes occur over narrow temperature intervals. As a result, the RC remains relatively low, particularly after accounting for magnetic and thermal hysteresis losses.

**Table 2.** Maximum entropy change, $\left|\Delta S_{M}^{\max}\right|$, Curie temperature, *T*_C_, refrigerant capacity (*RC*), and relative cooling power (*RCP*) for magnetocaloric thin film samples.

Samples	*T*_C_ (K)	µ_0_∆*H* (T)	$\left|\Delta S_{M}^{\max}\right|$ J/kg K)	*RC* (J/kg)	*RCP* (J/kg)	Ref.
***Gadolinium and its alloys***						
Gd (B)	294	1 2 5	2.80 5.07 10.20	–	63.4 187.0 410.0	[7]
Gd (F, *t* = 17μm)	292	1 3 5 7	2.70 5.90 8.30 10.50	~134.6 ~309.1 ~452.5 ~608.6	~140.2 ~308.6 ~452.2 ~627.5	[97]
Gd (F, *t* = 30 nm) As-deposited Annealed at 450 K	265 292	1 1	0.60 1.70	–	20.4 110.5	[25]
GdSi_2_ (F, *t* = 20 nm)	122	5	22.5	–	–	[125]
Gd_5_Si_2_Ge_2_ (B)	276	5	18.4	360	–	[45] (*)
Gd_5_Si_1.3_Ge_2.7_ (F, *t* = 780 nm)	193.5 (*T*_MS_)	5	8.83	212.0	–	[100] (*)
Gd_5_Si_1.3_Ge_2.7_ (F, *t* = 763 nm) Gd_5_Si_1.3_Ge_2.7_ Thermal cycling, 50 cycles Gd_5_Si_1.3_Ge_2.7_ Thermal cycling, 200 cycles Gd_5_Si_1.3_Ge_2.7_ Thermal cycling, 250 cycles Gd_5_Si_1.3_Ge_2.7_ Thermal cycling, 450 cycles Gd_5_Si_1.3_Ge_2.7_ Thermal cycling, 1000 cycles	192.5 192.5 192.5 192.5 192.5 192.5	5 5 5 5 5	8.10 7.34 6.96 6.71 1.52	156.8–142.7 N/A	–	[101] (*)
Gd_100-x_Co_x_ (F, *t* = 100 nm, *x* = 0–56) Gd_100_ Gd_60_Co_40_ Gd_56_Co_44_ Gd_52_Co_48_ Gd_48_Co_52_ Gd_44_Co_56_	280 190 205 239 282 337	2 2 2 2 2 2	1.97 2.51 2.64 1.99 1.71 1.27	–	106 139 158 139 152 148	[99]
Gd_x_(Fe_10_Co_90_)_100−x_ (F, *t* = 90 nm, *x* = 30–70) Gd_30_(Fe_10_Co_90_)_70_ Gd_40_(Fe_10_Co_90_)_60_ Gd_50_(Fe_10_Co_90_)_50_ Gd_55_(Fe_10_Co_90_)_45_ Gd_70_(Fe_10_Co_90_)_30_	*T*_comp_ = 436 540 508 558 598	1.5 1.5 1.5 1.5 1.5	0.25 0.48 0.97 0.86 0.75	–	-~28.8 ~58.2 ~51.6-	[126]
Pt/GdFeCo/Pt (F, *t* = 80 nm)	586.8 324.1 *(T*_comp_)	1.5	1.09	38.8 @ 560 K	–	[127]
Ta/GdFeCo/Ta (F, *t* = 80 nm)	664.3 389.7 *(T*_comp_)	1.5	0.78	15.84 @ 610 K	–	[127]
***Heusler alloys***						
Ni_53.4_Mn_33.2_Sn_13.4_ (F) *t* = 360 nm *t* = 700 nm *t* = 1000 nm	557 569 570	1.8 1.8 1.8	0.4375 1.025 1.188	0.9 2.375 2.65	–	[102]
Ni_53.2_Mn_29.2_Co_7.0_Sn_10.6_ (F) *t* = 360 nm *t* = 700 nm *t* = 1000 nm	860 863 866	1.8 1.8 1.8	0.165 0.176 0.187	7.85 9.21 9.45	–	[102]
Ni_53.5_Mn_23.8_Ga_22.7_ (F, *t* = 400 nm)	346	6	8.5	–	–	[128]
Ni_51_Mn_29_Ga_20_ (F, *t* = 250 nm)	355	0.5	1.4	–	–	[129]
Ni_48_(Co_5_)Mn_35_In_12_ (F, *t* = 200 nm)	353	9	8.8	–	–	[130]
Ni_51.6_Mn_32.9_Sn_15.5_ (F, *t* = 200 nm)	250	1	1.6	–	–	[131]
Ni_51_Mn_29_Ga_20_ (F, *t* = 250 nm) *Magnetostructural transition*	356	0.5	1.4 Cooling 1.0 Heating	15.4 Cooling 15.9 Heating	–	[5]
Ni_53.5_Mn_23.8_Ga_22.7_ (F, *t* = 400 nm)	346	6	8.5	~65	~76.5	[4]
Ni_43_Mn_32_Ga_20_Co_5_ (F, *t* = 350 nm)	340	2	~3.5	~60	~70	[132]
***Oxides***						
La_2/3_Ca_1/3_MnO_3_ (F, *t* = 260 nm) La_2/3_Ca_1/3_Mn_0.94_Cr_0.06_O_3_ (F, *t* = 260 nm) on LAO	228 ~193	1 1	8.5 0.83	~346 ~39	~255 ~23	[133]
La_0.7_Ca_0.3_MnO_3_ *B* La_0.7_Ca_0.3_MnO_3_ *F*; *t* = 150 nm	264 235	5 5	7.70 2.75	~187 ~200	–	[52] (*)
La_0.7_Ca_0.3_MnO_3_ *F*; *t* = 30 nm; Intrinsic *F*; *t* = 30 nm; Extrinsic	225 190	5 5	0.7 9	-~18	-~18	[103]
La_0.8_Ca_0.2_MnO_3_/STO (tensile strain) *t* = 25 nm *t* = 50 nm *t* = 75 nm *t* = 100 nm *t* = 300 nm La_0.8_Ca_0.2_MnO_3_/LAO (compressive) *t* = 25 nm *t* = 50 nm *t* = 75 nm *t* = 100 nm *t* = 300 nm	178 186 195 193 210 205 213 220 215 240	6 6 6 6 6 6 6 6 6 6	8.20 8.40 12.80 4.80 2.75 2.25 2.63 3.25 5.95 3.00	183 221 255 85 80 125 160 258 75 50	250 295 361 125 105 180 225 339 105 95	[106] [106]
La_0.88_Sr_0.12_MnO_3_ (F, *t* = 100 nm) La_0.88_Sr_0.12_MnO_3_ (F, *t* = 160 nm) La_0.88_Sr_0.12_MnO_3_ (F, *t* = 200 nm)	175 154 144	3 3 3	1.5 1.8 1.7	172 189 199	–	[104]
La_0.67_Sr_0.33_MnO_3_ (F, *t* = 20 nm) on LSAT La_0.67_Sr_0.33_MnO_3_ (F, *t* = 20 nm) on STO	321 312	1.5 1.5	1.47 1.54	–	32.24 50.16	[105]
La_0.67_Ba_0.33_Mn_0.95_Ti_0.05_O_3_ (F, *t* = 97 nm)	234	5	2.6	–	210	[134]
Pr_0.7_Sr_0.3_MnO_3_/PSMO-7 (F, *t* = 20 nm)	193	2	4.7	~131.6	~164.5	[135]
EuTiO_3_ (F, *t* = 100 nm)	3	2	24	152	–	[112]
Gd_2_NiMnO_6_ (F, *t* = 15 nm) In-plane Out-of-plane	125 11 11	5 5 5	1.40 21.82 9.84	–	75–	[120]
La_2_NiMnO_6_ (F, *t* = 200–250 nm) La_2_NiMnO_6_ (300 mTorr) La_2_NiMnO_6_ (100 mTorr) La_2_NiMnO_6_ (200 mTorr) La_2_NiMnO_6_ (300 mTorr) La_2_NiMnO_6_ (400 mTorr)	265 265 265 237.5 237.5 250 265	3 5 7 5 5 5 5	1.10 1.60 2.10 0.50 0.90 1.65 1.60	55 100 145 33.3 50 100 125	73.3 133.3 193.3–133.3-	[136]
GdCoO_3_/LAO (F, *t* = 22 nm)	3.5 (*T*_N_)	2 7	12.79 58.65	~31.0 319.8	~19.2 ~429.0	[115]
Eu$O_{1-\delta }$ (δ = 0, 0.025, 0.09) EuO_1_ (F, *t* = 100 nm) EuO_0.975_ (F, *t* = 100 nm) EuO_0.91_ (F, *t* = 100 nm)	69 118 133	2 2 2	6.2 7.1 5.1	460 760 670	525 880 780	[110]
PrVO_3_/LAO (F, *t* = 55 nm) PrVO_3_/STO (F, *t* = 100 nm) PrVO_3_/LSAT (F, *t* = 41.7 nm)	125 (*T*_N_) 125 (*T*_N_) 125 (*T*_N_)	5 5 5	0.44 0.26 0.31	16.24 11.36 13.27	20.4 13.4 15.7	[137]
***Others***						
SmCo_3_B_2_ (F, as-deposited) *t* = 90 nm *t* = 160 nm *t* = 240 nm	40 41 43	5 5 5	0.614 0.892 0.537	–	~2.3 ~2.4 ~2.7	[138]
SmCo_3_B_2_ (F) *t* = 90 nm *t* = 160 nm *t* = 240 nm	43 44 46	5 5 5	0.629 0.886 1.172	–	~2.7 ~3.1 ~5.3	[138]
Ta(20 nm)/Er-Co-Al(200-300 nm)/Ta(25 nm) ErCo_1.52_Al_0.36_ (as-deposited) ErCo_1.69_Al_0.76_ (as-deposited) ErCo_1.87_Al_0.16_ (as-deposited) ErCo_1.52_Al_0.36_ (annealed at 1073 K) ErCo_1.69_Al_0.76_ (annealed at 1073 K) ErCo_1.87_Al_0.16_ (annealed at 1073 K)	28 17.5 17.5 12.5 12.5 12.5	5 5 5 5 5 5	1.9 2.9 0.25 3.0 2.4 3.2	17.1 26.1 2.25 27 21.6 28.8	22.8 34.8 3.0 36 28.8 38.4	[139]
Tb_30_Fe_7_Co_63_ (F, *t* = 100 nm) *p* = 50 W *p* = 60 W *p* = 70 W *p* = 80 W *p* = 90 W *p* = 100 W	*T*_comp_ = 407 357 330 306 252 224	1.5 1.5 1.5 1.5 1.5 1.5	0.21 0.20 0.18 0.16 0.15 0.13	–	–	[140]
Epitaxial Tb (F, *t* = 100 nm) H//a axis (in-plane) H//b axis (in-plane) H//c axis (out-of-plane) Amorphous Tb (F, *t* = 100 nm) In-plane Out-of-plane	232 232 232 227 227	2 2 2 2 2	6.27 5.61 1.11 1.98 0.67		225 199 18 86 25	[121] [121]
(Fe_70_Ni_30_)_96_Mo_4_ (F, *t* = 30 nm)	323	1 2	0.77 1.38	119 228	–	[141]
CrF_3_ (2D van der Waals) (F, *t* = 5.19 nm)	18	5	32.2	–	–	[111]
CrCl_3_ (F, *t* = 6.06 nm)	22	5	21.9	–	–	[111]
CrBr_3_ (F, *t* = 6.44 nm)	36	5	12.5	–	–	[111]
CrI_3_ (F, *t* = 7.01 nm)	48	5	7.5	–	–	[111]
CrO_2_/TiO_2_ (F, *t* = 500 nm)	385	5	8.46	410 143 (1.5T)	–	[142]
Fe_2_Ta (F, *t* ~ 100 nm)	12.5 270	0.5	5.43x10^−4^ −1.58x10^−4^	–	–	[143] (*)
MnCoAs (F, *t* = 3.58 nm)	214–221	1–7	1.4–4.3	28.4–244.5	–	[144]
Fe_3_[Cr(CN)_6_]_2_⋅zH_2_O (F, *t* = 1400 nm)	20	1 5	3.2 10	21.1 273	–	[145]
Cr_3_[Cr(CN)_6_]_2_⋅zH_2_O (F, *t* = 1100 nm)	219	1 5	0.20 0.72	8 44	–	[145]
***Heterostructure and multi-layer structures***						
Py/Gd/CoFe/IrMn stacks Py = Ni_80_Fe_20_ Gd_thick_ = 20 nm	120–130	5	0.0256	–	–	[146]
Py/Gd/CoFe/IrMn stacks Py = Ni_80_Fe_20_ Gd_thick_ = 5 nm	85	3	0.0128	–	–	[146]
La_1-x_Sr_x_MnO_3_ (F, *t* = 35 nm) (*x* = 0.12) La_1-x_Sr_x_MnO_3_ (F, *t* = 35 nm) (*x* = 0.25) La_1-x_Sr_x_MnO_3_ 12/25 La_1-x_Sr_x_MnO_3_ 25/12	170 295 170/300 170/300	3 295	0.2 0.21 0.09/0.09 0.09/0.09	12 11 14 15	–	[147]
Gd(30 nm)/W(5 nm)	280	3	2.8	–	–	[98]
Quart/Ni_80_Fe_20_(10 nm)/Ni_67_Cu_33_(d nm)/Co_90_Fe_10_(3 nm)/ Ir_20_Mn_80_(25 nm)/TiO *d* = 3–15 nm	~330	0.003	10–15	~40–85	~50–105	[148]
Si/Co_90_Fe_10_(20 nm)/Ni_72_Cu_28_(d nm)/ Co₄₀Fe₄₀B₂₀(15 nm)/TiO *d* = 5–20 nm	~360	0.003	37.10	~180–250	~222–297	[148]
BiFeO_3_(15 nm)/LSMO(40 nm) BiFeO_3_(50 nm)/LSMO(40 nm) BiFeO_3_(120 nm)/LSMO(40 nm) BiFeO_3_(140 nm)/LSMO(40 nm)	~280 ~240 ~260 ~220	0.02 0.02 0.02 0.02	10 ×10^−4^ 7 ×10^−4^ 3 ×10^−4^ 1.32 ×10^−4^	0.21 0.125 0.04 0.01	--	[118]
Cr/Py/Fe_30_Cr_70_(6 nm)/Py/FeMn/Cr (Py = Ni_80_Fe_20_); *t* = 50 nm Cr/Py/Cr/Py/FeMn/Cr (Py = Ni_80_Fe_20_); *t* = 50 nm	162 160	0.025 0.025	~0.06–0.08 0.024	--	--	[149]
FeRh/BaTiO_3_ (F, *t* = 40 nm)	351	2	17	~272	~340	[114]
Ni_80_Fe_20_/Ni_67_Cu_33_/Co_90_Fe_10_/Mn_80_Ir_20_ Spacer Ni_67_Cu_33_ (*t* = 7 nm) Spacer Ni_67_Cu_33_ (*t* = 10 nm) Spacer Ni_67_Cu_33_ (*t* = 21 nm)	260 250 200	0.002 0.002 0.002	0.0067 0.0076 0.0133	~70.1 ~63.2 ~21.8	71.26 ~66.7 ~24.1	[119]
Fe/Fe-Cr/Fe simulated (F, *t* = 6 nm)	~200–214	0.25–1	> 6.4	–	–	[150]
FM/AFM = MnF_2_/FM (F, *t* = 30 nm)	~67 (*T*_N_)	1	13	~225	~300	[151]
Fe/Gd/Fe (F, *t* = 15 nm)	~200	0.03	1.27x10^−3^	–	–	[152]

*B: Bulk; F: Film; t: thickness; P: Pressure; T*_*N*_ *= Néel temperature; T*_*comp*_ *= compensation temperature; T*_*MS*_: Magnetostructural transition temperature. (*) represents FOMT materials.

Manganese oxide thin films, like La_0.7_Ca_0.3_MnO_3_ (150 nm), also exhibit reduced *T*_*C*_ (235 K vs. 264 K bulk) and -Δ*S*_*M*_^max^ (2.75 J/kg·K vs. 7.7 J/kg·K), though *RC* (*RCP*) can improve due to broadened FM-PM transitions [52]. To enhance Δ*S*_*M*_, Moya *et al*. exploited interfacial strain coupling with BaTiO_3_ substrates [103]. A sharp Δ*S*_*M*_ peak, with -Δ*S*_*M*_^max^ ~9 J/kg·K, was achieved at ~ 200 K in a 30 nm La_0.7_Ca_0.3_MnO_3_ film, induced by the structural phase transition of BaTiO_3_ from the rhombohedral (R) to orthorhombic (O) structure at *T*_R-O_ ~200 K. However, due to the narrow temperature span (~2 K), *RCP* was limited (~18 J/kg). It is worth noticing here that the application of external strain to induce and control the extrinsic MCE in magnetic films underscores the multicaloric nature of the La₀.₇Ca₀.₃MnO₃/BaTiO₃ heterostructure, suggesting that the cooling efficiency of magnetic refrigerants can be significantly enhanced by simultaneously leveraging multiple external stimuli, such as magnetic fields, electric fields, and mechanical strain.

**Figure 6.**
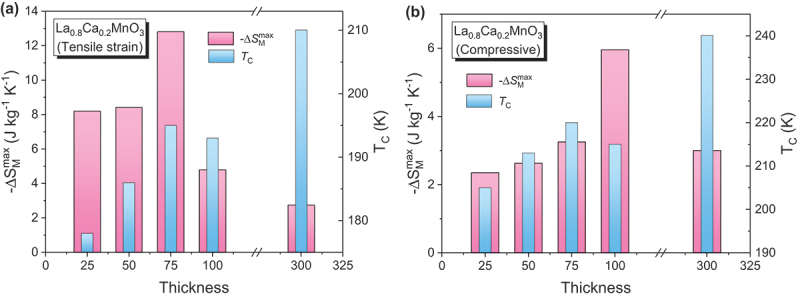
Variation of −Δ*S*_*M*_^*max*^ and *T*_*C*_ with the thickness of La_0.8_Ca_0.2_MnO₃ thin films under (a) tensile and (b) compressive strain, illustrating strain-induced changes in the magnetocaloric effect under a magnetic field change of 6 T.

Substrate-induced epitaxial strain plays a critical role in tuning magnetic and magnetocaloric responses [104–106]. In La_0.8_Ca_0.2_MnO_3_ films grown on SrTiO_3_ substrates, tensile strain reduces *T*_*C*_ from 210 K to 178 K with decreasing thickness, while enhancing -Δ*S*_*M*_^max^ (up to 12.8 J/kg·K) and *RC* (255 J/kg) at 75 nm (Figure 6(a)) [106]. Compressive strain (from LaAlO_3_ substrates) results in reduced -Δ*S*_*M*_^max^ and *RC* in La_0.8_Ca_0.2_MnO_3_ films (Figure 6(b)). These behaviors illustrate the complex interplay of film thickness, strain, and composition.

Oxygen non-stoichiometry is another key variable, contributing to discrepancies in reported -Δ*S*_*M*_^max^ and *RC* values [107–109]. Lampen-Kelley *et al*. showed that oxygen-deficient EuO_1–δ_ films (*δ* = 0–0.09) exhibit altered magnetic transitions and enhanced -Δ*S*_*M*_^max^ (up to 6.4 J/kg·K over 2 T) with broad refrigerant capacities (*RC* ~223 J/kg) [110]. Such tunability makes them promising for sub-liquid-nitrogen temperature applications. However, achieving precise control over the oxygen content in these and other manganese oxide thin films remains a significant challenge, particularly when tuning their magnetic and magnetocaloric properties.

In ultrathin films (few monolayers), quantum confinement effects may modify electronic states and exchange interactions. First-principles calculations by Patra *et al*. predict that 2D magnets like GdSi_2_ and Cr*X*_3_ (*X* = F, Cl, Br, and I) could exhibit substantial MCE at cryogenic temperatures, with -Δ*S*_*M*_^max^ as high as 22.5 J/kg·K [111]. However, experimental studies are needed to validate this prediction. It is worth noting that in atomically thin magnetic systems, the magnetic signals are typically weak and not easily detectable using standard magnetometry techniques. Consequently, accurately evaluating the MCE performance of these 2D materials is nontrivial and requires careful measurement and analysis.

For antiferromagnetic thin films, reducing thickness and introducing strain can weaken AFM interactions, sometimes inducing ferromagnetic behavior under moderate fields [111–114]. The effect is even more pronounced in mixed-phase films where AFM and FM phases coexist and coupled with each other [112,113]. In such systems, the application of a sufficiently strong magnetic field can induce a transition from AFM to FM order, leading to a significant change in magnetization and, consequently, a large magnetic entropy change, Δ*S*_*M*_. Zhou *et al*. reported a large -Δ*S*_*M*_^max^ of 20 J/kg·K at 320 K under a magnetic field change of 5 T for FeRh thin films, significantly outperforming their bulk counterpart (⁓12.6 J/kg·K) [33]. Owing to its FOMT nature, FeRh exhibits notable thermal and magnetic hysteresis losses, which can hinder its practical application. However, the incorporation of 3% and 5% Pd effectively shifts the Δ*S*_*M*_(*T*) peaks from 319 K to 281 K and 238 K, respectively, enabling better temperature tuning. The MCE behavior of FeRh thin films can vary significantly depending on the strain induced by the underlying substrate [113,114]. Furthermore, when FeRh is grown on a BaTiO₃ substrate, the application of an electric field can be used to modulate both the hysteresis losses and the MCE, making this multiferroic heterostructure a promising candidate for multicaloric cooling applications [114]. Bulk GdCoO₃ also exhibits AFM ordering originating from the Gd^3 +^ magnetic moments below its *T*_N_ of 3.1 K [153]. Under a magnetic field change of 7 T, it shows a large MCE with a -Δ*S*_*M*_^max^ of 39.1 J/kg·K, an Δ*T*_*ad*_ of 19.1 K, and a RC of 278 J/kg. This strong MCE arises from the half-filled 4f electronic configuration of Gd^3 +^ ions. When a 22 nm-thick GdCoO₃ thin film is epitaxially grown on a LaAlO₃ (LAO) substrate, the -Δ*S*_*M*_^max^ is further enhanced to ~59 J/kg·K, with an increased *RC* of ~320 J/kg around an elevated *T*_*N*_ of ~ 3.5 K for the same 7 T field change [115]. Similarly, 100 nm EuTiO_3_ films demonstrate -Δ*S*_*M*_^max^ ~24 J/kg·K and *RC* ~152 J/kg at ~ 3 K, compared to 17 J/kg·K and 107 J/kg in bulk under µ_0_*H* = 2 T [112]. These enhancements are attributed to strain effects and altered magnetic interactions at the nanoscale, demonstrating how finite-size and interfacial effects can amplify the MCE in antiferromagnetic thin film systems. Both GdCoO₃ and EuTiO₃ thin films hold strong potential as active cooling materials for NEMS and MEMS operating at cryogenic temperatures, owing to their enhanced magnetocaloric response at the nanoscale.

**Figure 7.**
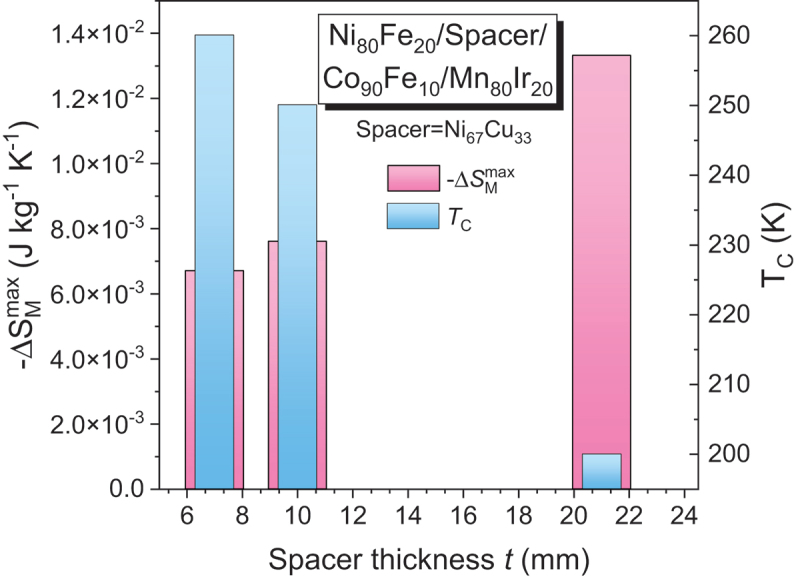
Dependence of −Δ*S*_*M*_^*max*^ and *T*_*C*_ on spacer thickness, highlighting the tunability of magnetocaloric behavior through layer design for Ni_80_Fe_20_/Spacer/Co_90_Fe_10_/Mn_80_Ir_20_ (Spacer = Ni_67_Cu_33_) under a magnetic field change of 2 mT.

Multilayer and heterostructure films (e.g. FM/NM or FM/AFM) also demonstrate interfacial effects like proximity-induced magnetism and exchange bias [116,117]. In BiFeO_3_/LSMO heterostructures, increasing AFM BiFeO_3_ layer thickness decreases LSMO’s *T*_*C*_ and Δ*S*_*M*_ [118] In Ni_80_Fe_20_/Ni_67_Cu_33_/Co_90_Fe_10_/Mn_80_Ir_20_ films, increasing spacer thickness of Ni_67_Cu_33_ decreases *T*_*C*_ but enhances -Δ*S*_*M*_^max^ and *RC* (see Figure 7), showing how interlayer coupling influences MCE [119].

In thin films exhibiting significant MCE anisotropy, magnetic entropy change can be triggered simply by rotating the material within a constant magnetic field, rather than switching the field on and off [120,121]. This ‘rotating MCE’ approach reduces energy losses associated with magnetic field cycling and enables simpler, more compact device architectures [122–124]. Understanding and harnessing MCE anisotropy is essential for selecting or engineering materials, such as layered structures or textured films, where the anisotropy can be tuned to maximize Δ*S*_*M*_ along specific crystallographic directions. For example, in Gd₂NiMnO₆ thin films, although the *T*_*C*_ remains unaffected by film orientation, -Δ*S*_*M*_^max^ varies significantly from 9.84 J/kg·K (out-of-plane) to 21.82 J/kg·K (in-plane), yielding a large rotating entropy change of 11.98 J/kg·K [120]. A similar directional dependence has also been observed in epitaxial Tb films [121].

Lastly, it is worth mentioning that thermal transport in thin films differs from bulk, affecting device-level performance of magnetic refrigeration systems. While thin films offer tunable MCE through dimensionality, strain, and interface engineering, practical challenges remain, especially in maximizing Δ*S*_*M*_ without sacrificing thermal efficiency or cyclic durability.

### 3.3. Ribbons

Magnetocaloric ribbons are typically fabricated using a rapid solidification technique known as melt spinning, which enables the formation of thin, amorphous and/or nanocrystalline ribbons with controlled microstructures [154–248]. In this process, molten metal is ejected onto a rotating copper wheel and solidifies almost instantaneously at cooling rates of approximately 10^6^ K/s, producing ribbons typically 20–50 μm thick and 1–5 mm wide. Depending on the targeted magnetic and structural properties, the ribbons may undergo post-annealing to induce nanocrystallization, promote the formation of desired magnetic phases, or relieve internal stresses. Such thermal treatments are particularly critical for amorphous ribbons, which often require structural tuning to enhance their magnetocaloric performance.

The base magnetocaloric alloys, such as Gd-based, Fe-based, LaFeSi-based, Heusler, or high-entropy alloys, are initially synthesized by arc melting or induction melting of high-purity elemental constituents under an inert argon atmosphere. The key magnetocaloric properties of these alloy ribbons are summarized in Table 3.

**Table 3.** Maximum entropy change, $\left|\Delta S_{M}^{\max}\right|$, Curie temperature, *T*_C_, refrigerant capacity (*RC*), and relative cooling power (*RCP*) for the ribbon samples.

Samples	*T*_C_ (K)	µ_0_∆*H* (T)	$\left|\Delta S_{M}^{\max}\right|$ (J/kg K)	*RC* (J/kg)	*RCP* (J/kg)	Ref.
***Gadolinium and its alloys***						
Gd (*B*)	294	1 2 5	2.8 5.07 10.2	––~400	63.4 187 410	[7]
Gd (*R*)	294	1.7	4.8	–	–	[176]
Gd (*R*)	293	5	8.7	433.4		[157]
Gd_71_Co_29_ (*R*; *A*)	166	1	3.1	92.3	–	[158]
Gd_68_Co_32_ (*R*; *A*)	175	1	3.0	87.4	–	[158]
Gd_65_Co_35_ (*R*; *A*)	184	1	2.9	83.6	–	[158]
Gd_62_Co_38_ (*R*; *A*)	193	1	2.8	81.4	–	[158]
Gd_48_Co_52_ (*R*; *A*)	282 282	1.5 5	1.71 4.23	--	176 750	[177]
Gd_4_Co_3_ (*R*; *A*)	219	5	7.2	–	–	[178]
Gd_60_Co_25_Al_15_ (*R*; *A*)	125	5	10.1	645	860	[160]
Gd_55_Co_25_Al_20_ (*R*; *A*)	112.5	5	10.1	612.6	818	[161]
Gd_60_Co_30_Al_10_ (*R*; *A*; Sheet parallel)	140	1.9	4.06	64	–	[179]
Gd_60_Co_30_Al_10_ (*R*; *A*; Sheet perpendicular)	140	1.9	2.91	34	–	[179]
Gd_50_Co_50-*x*_Fe_*x*_ (*R*; *A*) *x* = 0 *x* = 2	267 277	5 5	-4.44	--	--	[180]
Gd_50_Co_50-*x*_Si_*x*_ (*R*; *A*) *x* = 2 *x* = 5	214 244	5 5	5.32 5.98	--	710 740	[181]
Gd_48_Co_50_Zn_2_ (*R*; *A*)	262	5	5.02	–	> 700	[182]
Gd_50_Co_48_Zn_2_ (*R*; *A*)	260	5	5.04	–	700	[182]
Gd_55_Co_35_*M*_10_ (*R*; *A*) *M* = Mn *M* = Fe *M* = Ni	197 268 192	2 2 2	3.03 1.72 3.37	162 266 183	224 337 253	[183]
Gd_55_Co_20_Fe_5_Al_20-*x*_Si_*x*_ (*R*; *A*) *x* = 0 *x* = 5 *x* = 10 *x* = 15	130 142 149 151	5 5 5 5	6.1 6.82 6.36 4.94	558 665 700 519	–	[184]
Gd_55_Co_20_Fe_5_Al_20-*x*_Si_*x*_ (*x* = 0, 2, 5, 10) (*R*; *A*) (*x* = 15, 20, 20; annealed) (*R*; *C*)	129 136 108 137 130 130 –	5 5 5 5 5 5 5	8.01 6.66 4.90 4.48 4.77-4.78	–	913 719 541 622 596--	[185]
Gd_65_Fe_20_Al_15_ (*R*; *A*)	182	5	5.8	545	726	[162]
Gd_55_Fe_15_Al_30_ (*R*; *A*)	158	5	5.01	555	741	[162]
Gd_55_Fe_20_Al_25_ (*R*; *A*)	190	5	4.67	651	868	[162]
Gd_55_Fe_25_Al_20_ (*R*; *A*)	230	5	3.77	608	811	[162]
Gd_95_Fe_2.8_Al_2.2_ (*R*; *A*)	232	5	4	551	–	[186]
Gd_95_Fe_2.8_Al_2.2_ (*R*; *C*)	232	5	7.53	551	–	[186]
Gd_55_Fe_15_Al_30_ (*R*; *A*)	158	5	5.01	741	–	[162]
Gd_55_Fe_20_Al_25_ (*R*; *A*)	182	5	4.67	868	–	[162]
Gd_55_Fe_25_Al_20_ (*R*; *A*)	197	5	3.77	811	–	[162]
Gd_55_Fe_30_Al_15_ (*R*; *A*)	208	5	3.43	857	–	[162]
Gd_55_Fe_35_Al_10_ (*R*; *A*)	228	5	2.92	826	–	[162]
Gd_71_Fe_3_Al_26_ (*R*; *A*)	117.5	5	7.4	750	–	[187]
Gd_65_Fe_20_Al_15_ (*R*; *A*)	182.5	5	5.8	726	–	[187]
RNi (*R* = Gd, Tb and Ho) (*R*; *C*)	75 66 28	5 5 5	15.2 12 14.1	–	610 370 550	[188]
Gd_63_Ni_37_ (*R*; *A*)	122	5	9.42	600	802.6	[189]
Gd_71_Ni_29_ (*R*; *A*)	122	5	9	724	–	[159]
Gd_68_Ni_32_ (*R*; *A*)	124	5	8	583	–	[159]
Gd_65_Ni_35_ (*R*; *A*)	122	5	6.9	524	–	[159]
Gd_46_Ni_32_Al_22_ (*R*; *A*)	66	5	10.16	762	–	[190]
Gd_55_Ni_15_Al_30_ (*R*; *A*)	70	5	6.12	606	–	[163]
Gd_55_Ni_20_Al_25_ (*R*; *A*)	71	5	7.98	782	–	[163]
Gd_55_Ni_25_Al_20_ (*R*; *A*)	75	5	8.49	806	–	[163]
Gd_55_Ni_30_Al_15_ (*R*; *A*)	83	5	9.25	851	–	[163]
Gd_34_Ni_22_Co_11_Al_33_ (*R*; *A*)	54	5	9.9	–	145	[191]
Gd_100-*x*_Mn_*x*_ (*R*; *C*) *x* = 0 *x* = 5 *x* = 10 *x* = 15 *x* = 20	293 289 287 285 278	5 5 5 5 5	8.7 8.3 6.8 6.6 5.9	433.4 451.9 353.7 354.5 321.5	–	[157]
Gd_65_Mn_35-*x*_Si_*x*_ (*R*; *A*) *x* = 5 *x* = 10	221 218	5 5	4.6 4.7	625 660	--	[192]
Gd_65_Mn_25_Si_10_ (*R*; *A*+*C*)	288	5	4.6	249	–	[192]
(Gd_4_Co_3_)_1-*x*_Si_*x*_ (*R*; *A*) *x* = 0 *x* = 0.05 *x* = 0.10	208 198 213	5 5 5	7.3 7.2 6.4	547 524 511	–	[193]
(Gd_1-*x*_Tb_*x*_)_12_Co_7_ (*R*; *A*) *x* = 0 *x* = 0.25 *x* = 0.5 *x* = 0.75 *x* = 1	179 159 136 118 92	5 5 5 5 5	7.9 8.0 9.0 8.5 8.4	511 522 540 462 456	–	[194]
GdCuAl (*R*; *A*+*C*)	50	5	5.6	296	–	[195]
Gd_55_Co_25_Ni_20_ (*R*; *A*)	140	5	6.04	450	–	[196]
Gd_55_Co_30_Ni_15_ (*R*; *A*)	175	5	6.3	487	–	[196]
Gd_55_Co_35_Ni_10_ (*R*; *A*)	192	5	6.47	502	–	[196]
Gd_60_Mn_30_Ga_10_ (*R*; *A + C*)	177	2	1.53	240	–	[197]
Gd_60_Mn_30_In_10_ (*R*; *A+C*)	190	2	1.49	234	–	[197]
Gd_60_Co_30_In_10_ (*R*; *A+C*)	159	4.6	7.7	406	–	[198]
Gd_60_Ni_30_In_10_ (*R*; *A+C*)	86	4.6	8.2	602	–	[198]
Gd_60_Cu_30_In_10_ (*R*; *A+C*)	115	4.6	6.6	598	–	[198]
Gd_60_Fe_0_Co_30_Al_10_ (*R*; *A+C*)	145	5	8.9	539	–	[199]
Gd_60_Fe_10_Co_20_Al_10_ (*R*; *A+C*)	170	5	5	632	–	[199]
Gd_60_Fe_20_Co_10_Al_10_ (*R*; *A+C*)	185	5	4.4	736	–	[199]
Gd_60_Fe_30_Co_0_Al_10_ (*R*; *A+C*)	200	5	3.6	672	–	[199]
Gd_45_RE_20_Fe_20_Al_15_ (*R*; *A*) (RE = Tb, Dy, Ho, Er)	138- 175	5	4.46- 5.57	580- 720	–	[200]
Gd_55_Co_19_Al_24_Si_1_Fe_1_ (*R*; *A*+*C*)	107	5	7.8	749	–	[154]
Gd_55_Co_35_Mn_10_ (*R*; *A*+*C*) (50 m/s)	200 200	2 5	3.82 6.47	183.4 457.3	233.0 601.7	[201]
Gd_55_Co_35_Mn_10_ (*R*; *A*+*C*) (600 K/20 min)	123/173	2 5	2.93 5.50	233.5 515.7	284.2 649	[201]
Gd_55_Co_35_Mn_10_ (*R*; *A*+*C*) (600 K/30 min)	123/170	2 5	2.79 5.46	242.1 536.4	284.6 671.6	[201]
Gd_65_Fe_10_Co_10_Al_15_ (*R*; *A*)	160	5	6.0	700	–	[202]
Gd_65_Fe_10_Co_10_Al_10_Si_5_ (*R*; *A*)	175	5	5.9	698	–	[202]
Gd_65_Fe_10_Co_10_Al_10_B_5_ (*R*; *A*)	145	5	7.1	748	–	[202]
Gd_55_Co_35_Ni_10_ (*R*; *A*)	158/214	5	5.0	–	–	[203]
Gd_50_Co_45_Fe_5_ (*R*; *A*)	289	5	3.8	–	673	[204]
Tm_60_Al_20_Ni_10_ (*R*; *A*)	4.4	5	14.1	–	235	[205]
Er_60_Al_20_Ni_10_ (*R*; *A*)	9.5	5	14.3	–	372	[205]
Ho_60_Al_20_Ni_10_ (*R*; *A*)	17.9	5	12.4	–	460	[205]
ErNi_2_ (*R*; *C*)	6.8	2 5	14.1 20.0	146 382	–	[206]
ErNi_2_ (*R*; *C*)	6.8	2 5	12.4 20.2	118 347	–	[206]
TbNi_2_ (*R*; *C*)	37	5	13.9	441	–	[207]
DyNi_2_ (*R*; *C*)	21.5	2	13.5	209	–	[208]
HoNi_2_ (*R*; *C*)	13.9 13.9	2 5	16.9 27.2	194 522	–	[209]
HoNi_2_ (*R*; *C*)	13.9 13.9	2 5	14.8 24.8	169 465	–	[209]
Dy_3_Co (*R*; *C*)	32 43	2 5	2.1 6.5	--	83 364	[210]
Tb_55_Co_30_Fe_15_ (*R*; *A*)	169	5	4	–	–	[211]
***Heusler alloys***						
Mn_50_Ni_41_In_9_ (*R*; *C*)	283	3	5.7	184.2	197.8	[212]
Mn_50_Ni_40_In_10_, *H*∥ Melt-spun ribbons, Mn_50_Ni_40_In_10_, *H*⊥ Melt-spun ribbons (*R*; *C*)	230 310 230 310	3 3	3.6 1.3 3.5 1.3	71 89 71 86	--	[213] (*) (*)
Ni_50_Mn_50-x_Sn_x_ (*R*; *C*) *x* = 13	255 300	5	22 4	160 75	–	[19] (*)
Ni_52_Mn_26_Ga_22_ (*R*; *C*) As-melt Annealed	350 354	2 5 2 5	5.3 11.4 16.4 30	--32 70	--	[214]
Mn_3_Sn_2-x_B_x_ (*R*; *C*) (*x* = 0–0.5)	240–250	5	13.6–18.3	–	–	[215]
Mn_3_Sn_2-x_C_x_ (*R*; *C*) (*x* = 0–0.5)	240–250	5	13.6–17.5	–	–	[215]
Ni_51.1_Mn_31.2_In_17.7_ (*R*; *C*) Annealed at 1073 K/10 min Annealed at 1073 K/2 h	276 288 288	5 5 5	3.1 4.1 4.4	345 268 294	–	[216]
Ni_43_Mn_46_In_11_ (*R*; *C*) Slow cooled Quenched	245 304 260 320 263 311	5 5 5	1.31 1.45 3.48 2.05 6.79 2.72	--	38.5 95.7 97.4 114.8 142.6 152.3	[217] (*) (*) (*)
Ni_52_Mn_26_Ga_22_ (*R*; *C*)	348 348	2 5	5.3 11.4	--	--	[218]
Mn_50_Ni_40.5_In_9.5_ (*R*; *C*)	295	5	3.7	80.5	–	[219]
Mn_50_Ni_40.5_In_9.5_ (*R*; *C*, Annealed)	326	5	6.1	126.6	–	[219]
Ni_45_Co_5_Mn_31_Al_19_ (*R*; *C*)	265 291	1.35	2 1	–	–	[220] (*)
Ni_46_Co_4_Mn_38_Sb_12_ (*R*; *C*) (In-plane)	297	5	13.5 12.6	–	–	[221]
Ni_46_Co_4_Mn_38_Sb_12_ (*R*; *C*) (Out of plane)	297	5	12.6	–	–	[221]
Ni_42.9_Co_6.9_Mn_38.3_Sn_11.9_ (*R*; *C*)	302	1	6.7	45.3	–	[222]
Ni_42.9_Co_6.9_Mn_38.3_Sn_11.9_ (*R*; *C*; Annealed)	308	1	25.3	55.8	–	[222]
Ni_50_Mn_35_In_14.25_B_0.75_ (*R*; *C*)	318	5	16	85	–	[223]
Ni_48_Co_2_Mn_35_In_15_ (*R*; *C*)	326	5	12.1	78	–	[224]
Ni_50−x_Co_x_Mn_35_In_15_ (*R*; *C*) *x* = 0 *x* = 1 *x* = 2	305 315 325	1 1 1	0.92 5.35 3.90	6.97 31.87 42.05	–	[225]
Ni_45_Co_5_Mn_40_Sn_10_ (*R*; *C*)	436 431 426 424	1 3 5 7	2 10 22 27	--	--	[226]
Ni_42.7_Mn_40.8_Co_5.2_Sn_11.3_ (*R*; *C*)	263 377.5	5	6.8 1.3	24 67	–	[227] (*)
Ni_42.7_Mn_40.8_Co_5.2_Sn_11.3_ (*R*; *C*) annealed at 1123 K/10 min	270 379	5	32.8 1.6	44 91	–	[227] (*)
***Fe-based alloys***						
Fe_90_Zr_10_ (*R*; *A*)	230	2 5 8	1.3 2.7 3.9	194 497 801	–	[165]
Fe_90_Zr_9_B_1_ (*R*; *A*)	210	2 5 8	1.3 2.7 3.8	198 492 795	–	[165]
Fe_91_Zr_7_B_2_ (*R*; *A*)	215	2 5 8	1.2 2.5 3.6	177 462 755	–	[165]
Fe_90_Zr_8_B_2_ (*R*; *A*)	240	2 5 8	1.3 2.6 3.7	198 514 830	–	[165]
Fe_88_Zr_8_B_4_ (*R*; *A*)	280	2 5 8	1.3 2.8 4.0	201 551 905	–	[165]
Fe_87_Zr_6_B_6_Cu_1_ (*R*; *A*)	300	2 5 8	1.6 3.0 4.3	208 590 953	–	[165]
Fe_86_Zr_7_B_6_Cu_1_ (*R*; *A*)	320	2 5 8	1.6 3.1 4.4	205 582-	–	[165]
Fe_89_Zr_7_B_4_ (*R*; *A*)	275	5	3.19	–	–	[228]
Fe_87_Zr_7_B_4_Dy_2_ (*R*; *A*)	308	5	3.14	–	–	[228]
Fe_87_Zr_7_B_4_Tb_2_ (*R*; *A*)	319	5	3.25	–	–	[228]
Fe_87_Zr_7_B_4_Gd_2_ (*R*; *A*)	342	5	3.24	–	–	[228]
Fe_89_Zr_8_B_3_ (*R*; *A*)	271	5	2.75	–	–	[229]
Fe_88_Zr_8_B_4_ (*R*; *A*)	291	5	3.04	–	644.9	[229]
Fe_87_Zr_8_B_5_ (*R*; *A*)	306	5	3.25	–	–	[229]
Fe_88_Zr_9_B_3_ (*R*; *A*)	286	5	3.17	–	686.7	[230]
Fe_87_Zr_9_B_4_ (*R*; *A*)	304	5	3.29	–	–	[230]
Fe_86_Zr_9_B_5_ (*R*; *A*)	327	5	3.34	–	–	[230]
Fe_88_Gd_2_Zr_10_ (*R*; *A*)	285	5	4.03	–	282	[231]
Fe_65_Mn_15_B_20_ (*R*; *A*)	328	1.5	0.89	72.5	99.7	[232]
Fe_60_Mn_20_B_20_ (*R*; *A*)	200	1.5	0.6	62.5	84.5	[232]
Fe_56_Mn_24_B_20_ (*R*; *A*)	170	1.5	0.55	51	66.7	[232]
Fe_70_Mn_10_B_20_ (*R*; *A*)	450	1.5	1.01	84.4	117	[232]
Fe_80_Cr_8_B_12_ (*R*; *A*)	328	1.5	1.00	–	130	[233]
Fe_88_Zr_7_B_4_Ni_1_ (*R*; *A*)	285	1.5 5	1.32 3.24	--	132-	[234]
Fe_88_Zr_7_B_4_Al_1_ (*R*; *A*)	280	1.5	1.37	–	–	[234]
Fe_88_Zr_9_B_1_Co_2_ (*R*; *A*)	285	1.5	1.61	–	149.7	[235]
Fe_87_Zr_11_B_1_Co_1_ (*R*; *A*)	280	1.5	1.38	–	133.9	[235]
Fe_88_Ce_7_B_5_ (*R*; *A*)	287	1.5 5	1.52 3.83	--	-700.9	[236]
Fe_88_La_2_Ce_5_B_5_ (*R*; *A*)	293	1.5 5	1.53 3.85	--	-656.7	[236]
Fe_90−x_Ni_x_Zr_10_ (*R*; *A*) *x* = 0 *x* = 5 *x* = 10 *x* = 15	245 306 356 403	4 4 4 4	3.04 3.26 3.30 3.10	334 290--	--	[166]
Fe_90-x_Sn_x_Zr_10_ (*R*; *A*) *x* = 0 *x* = 2 *x* = 4	247 269 293	5 5 5	3.6 4.1 3.4	320 255 228	410 337 280	[237]
Fe_82_B_4_Mn_4_Zr_8_Nb_2_ (*R*; *A*)	237	1 3	0.97 2.19	–	–	[238]
Fe_78_B_8_Mn_4_Zr_8_Nb_2_ (*R*; *A*)	259	1 3	0.88 1.97	–	–	[238]
Fe_74_B_12_Mn_4_Zr_8_Nb_2_ (*R*; *A*)	282	1 3	0.73 1.63	–	–	[238]
Fe_70_B_16_Mn_4_Zr_8_Nb_2_ (*R*; *A*)	313	1 3	0.68 1.58	–	–	[238]
Fe_66_B_20_Mn_4_Zr_8_Nb_2_ (*R*; *A*)	328	1 3	0.62 1.38	–	–	[238]
Fe_64_Mn_16_P_10_B_7_C_3_ (*R*; *A*)	266	1.5 2	0.78 0.98	74.7 101.5	101.05 139.74	[239]
Fe_65_Mn_15_P_10_B_7_C_3_ (*R*; *A*)	292	1.5 2	0.91 1.12	79.8 109.2	117.53 147.09	[239]
Fe_66_Mn_14_P_10_B_7_C_3_ (*R*; *A*)	319	1.5 2	0.91 1.12	71.9 99.8	99.84 134.25	[239]
Fe_67_Mn_13_P_10_B_7_C_3_ (*R*; *A*)	339	1.5 2	1.00 1.24	67.2 93.4	90.07 127.57	[239]
Fe_88_Zr_7_B_4_Cu_1_ (*R*; *A*)	287	1.5	1.32	121	166	[240]
Fe_82.5_Co_2.75_Ni_2.75_Zr_7_B_4_Cu_1_ (*R*; *A*)	400	1.5	1.4	119	165	[240]
Fe_78_Co_5_Ni_5_Zr_7_B_4_Cu_1_ (*R*; *A*)	500	1.5	1.85	95	125	[240]
Fe_71.5_Co_8.25_Ni_8.25_Zr_7_B_4_Cu_1_ (*R*; *A*)	570	1.5	1.95	97	130	[240]
Fe_66_Co_11_Ni_11_Zr_7_B_4_Cu_1_ (*R*; *A*)	640	1.5	1.80	98	131	[240]
Fe_88_Pr_6_Ce_4_B_2_ (*R*; *A*)	284	5	4.15	–	725.8	[241]
Fe_87_Zr_7_B_4_Co_2_ (*R*; *A*)	333	5	3.42	–	–	[242]
Fe_62_Mn_18_P_10_B_7_C_3_ (*R*; *A*)	222	1.5 2	0.57 0.71	48 67.2	64.57 87.68	[239]
Fe_60_Co_12_Gd_4_Mo_3_B_21_ (*R*; *A*)	387	1	0.76	–	–	[243]
***Intermetallic compounds***						
Nd_2_Fe_17_ (*R*; *C*)	326	1 2 3 4 5	1.4 2.5 3.3 4.1 4.8	–	73 169 271 382 496	[155]
Y_2_Fe_17_ (*R*; *C*)	301	1 2 5	1.5 2.4 4.4	–	75 178 533	[172]
Y_2_Fe_17_ (*R*; *C*)	305	10	1.89	–	–	[173]
Pr_2_Fe_17_ (*R*; *C*)	290	1 2 3 4 5	1.0 1.8 2.5 3.1 3.7	–	95 208 328 450 580	[155]
NdPrFe_17_ (*R*; *C*)	303/332	2	2.1	175	–	[244]
Pr_2-x_Nd_x_Fe_17_ (*R*; *C*) *x* = 0.5 *x* = 0.7	302 307	5 5	3.01 4.31	--	345 487	[174]
LaFe_12_Si (*R*; *C*) Annealed at 1323 K/2 h	195	5	25.4 @ 201 K	–	–	[245] (*)
LaFe_11.8_Si_1.2_ (*R*; *C*) Annealed at 1323 K/2 h	195	5	31 @ 201 K	–	–	[245] (*)
LaFe_11.2_Si_1.8_ (*R*; *C*) Annealed at 1323 K/2 h	231	5	10.3 @ 240 K	–	–	[245] (*)
LaFe_11.5_Si_1.5_ (*R*; *C*) Annealed at 1273 K/0.033 h	189	5	12	–	–	[171] (*)
LaFe_11.5_Si_1.5_ (*R*; *C*) Annealed at 1273 K/2 h	201	5	17	–	–	[171] (*)
LaFe_11.6_Si_1.4_ (*R*; *C*) Annealed at 1373 K/24 h	199	5	10.03	–	–	[171] (*)
LaFe_11.6_Si_1.4_ (*R*; *C*) Annealed at 1323 K/0.5 h	223	5	6.30	–	–	[171]
LaFe_11.6_Si_1.4_ (*R*; *C*) Annealed at 1323 K/4 h	213	5	8.13	–	–	[171]
LaFe_11.57_Si_1.43_ (*R*; *C*) Annealed at 1323 K/2 h	210	5	21.2	–	–	[246] (*)
LaFe_11.57_Si_1.43_ (*R*; *C*) Annealed at 1273 K/1 h (20 m/s)	198	5	17.8	–	–	[246] (*)
LaFe_11.57_Si_1.43_ (*R*; *C*) Annealed at 1273 K/1 h (40 m/s)	210	5	193	–	–	[246] (*)
LaFe_11.6 × 1.1_Si_1.4_ (*R*; *C*) Annealed at 1523 K/5 h	190.5	5	17.2	–	146.2	[246] (*)
LaFe_11.6 × 1.2_Si_1.4_ (*R*; *C*) Annealed at 1523 K/5 h	177.4	5	13.2	–	105.6	[246] (*)
La_0.8_Ce_0.2_Fe_11.5_Si_1.5_ (*R*; *C*) Annealed at 1273 K/10 mins Annealed at 1273 K/15 mins Annealed at 1273 K/20 mins Annealed at 1273 K/30 mins Annealed at 1273 K/60 mins	193 188 183 184 183	1.5 1.5 1.5 1.5 1.5	9.7 23 33.8 31.4 32.8	–	–	[247] (*)
La_0.6_Pr_0.5_Fe_11.4_Si_1.6_ (*R*; *C*)	192	5	21.9	458.5	481.8	[248] (*)
***High entropy alloys (HEAs)***						
Tm_10_Ho_20_Gd_20_Ni_20_Al_20_ (*R*; *A*)	30.3	3 7	5.6 12.7	223.4 637.4	282.9 793.5	[175]
Gd_20_Dy_20_Er_20_Co_20_Al_20_ (*R*; *A*)	42	5	7.7	523	–	[154]

R: Ribbon; A: Amorphous; C: Crystalline; B: Bulk; (*) represents FOMT materials.

**Figure 8.**
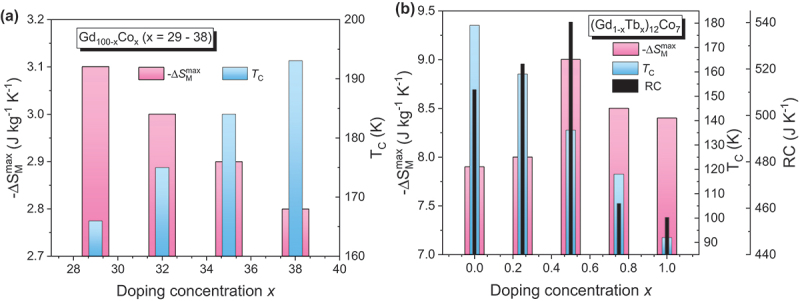
(a) Maximum magnetic entropy change (-Δ*S*_*M*_^max^) and Curie temperature (*T*_*C*_) as functions of Co doping concentration (*x*) in Gd_100−x_Co_x_ ribbons under a field of 1 T; (b) *T*_*C*_, -Δ*S*_*M*_^max^, and *RC* as functions of Tb doping concentration (*x*) in (Gd_1−x_Tbx)₁₂Co₇ alloys under a field of 5 T.

Compared to bulk Gd (-Δ*S*_*M*_^max^ ~10.2 J/kg·K and *RC* ~400 J/kg at µ₀*H* = 5 T) [7], its ribbon counterpart exhibits a slightly reduced magnetic entropy change (-Δ*S*_*M*_^max^ ~8.7 J/kg·K) but a modestly enhanced refrigerant capacity (*RC* ~433 J/kg), while maintaining a *T*_*C*_ near 294 K [157]. Alloying strategies have been employed to tune the magnetocaloric properties of Gd-based ribbons [157–159]. For examples, Gd – Co alloys show an increase in *T*_*C*_, but at the expense of reduced -Δ*S*_*M*_^max^, as can be seen in Figure 8(a) [158]. Gd – Ni alloys retain *T*_*C*_ close to that of pure Gd but still exhibit a reduction in -Δ*S*_*M*_^max^ [159]. In Gd_100–x_Mn_x_ ribbons, both *T*_*C*_ and -Δ*S*_*M*_^max^ decrease with increasing Mn content [157]. Interestingly, while Gd – Mn ribbons generally display higher Δ*S*_*M*_^max^ and *RC* than their Gd – Co counterparts, the latter maintain higher *T*_*C*_ values. The incorporation of Al into Gd – Co alloys has been found to enhance the MCE, albeit with a further reduction in *T*_*C*_ [160,161]. In Gd – Fe – Al ribbons, increasing the Fe/Al ratio leads to higher *T*_*C*_ but a decrease in -Δ*S*_*M*_^max^ [162]. Conversely, in Gd – Ni – Al systems, a higher Ni/Al ratio has been reported to simultaneously increase both *T*_*C*_ and -Δ*S*_*M*_^max^ [163]. For (Gd_1-x_Tb_x_)_12_Co_7_ ribbons, substituting Tb for Gd decreases *T*_*C*_, but the highest values of -Δ*S*_*M*_^max^ and *RC* are achieved at *x* = 0.5 (see Figure 8(b)). Overall, alloying Gd with multiple elements tends to either raise *T*_*C*_ while lowering -Δ*S*_*M*_^max^, or vice versa. Only a limited number of Gd-based alloy ribbons maintain *T*_*C*_ values near ambient temperature, which is a key requirement for room-temperature magnetic refrigeration.

To enable high-temperature magnetic cooling, the magnetocaloric properties of various Heusler alloy ribbon systems have been investigated (Table 3) [19,212–227]. In Heusler alloys, the magnetization and its variation associated with the martensitic transition are strongly influenced by the valence electron concentration per atom (e/a), which can be effectively modulated through chemical doping with elements such as Fe, Co, Cu, In, and Ge. As a result, both *T*_*C*_ and Δ*S*_*M*_ of these alloys can be finely tuned over a broad temperature range. Most Heusler alloy ribbons exhibit SOMT ferromagnetic ordering at or above room temperature, followed by a FOMT at lower temperatures [19]. Notably, larger Δ*S*_*M*_ values are typically observed around the FOMT, albeit within a narrower temperature window. In contrast, Δ*S*_*M*_ values around the SOMT are generally smaller but extend over a wider temperature range. Consequently, some Heusler alloy systems exhibit larger *RCs* around the SOMT (see Table 3). However, significant hysteretic losses are often reported in these systems, particularly associated with the FOMT, which can substantially reduce the *RC* [19,213]. By carefully refining the chemical composition, it is possible to minimize these hysteretic losses, thereby enhancing the *RC* while retaining the high MCE values. Specialized thermal treatment is also essential for optimizing the MCE performance in these Heusler alloy ribbons [164,216,219].

Fe-based magnetocaloric ribbons have also been widely studied for their promising MCE characteristics [165,166,228–243]. For instance, Fe₉₀Zr₁₀ ribbons exhibit a -Δ*S*_*M*_^max^ of approximately 2.7 J/kg·K and a *RC* of 497 J/kg under a 5 T magnetic field [165]. The incorporation of 1–2% boron (B) into this alloy tends to reduce both the *T*_*C*_ and -Δ*S*_*M*_^max^ [165]. However, careful adjustment of the Fe – Zr – B composition can simultaneously enhance both parameters. Notably, the addition of 1% Cu to form Fe₈₆Zr₇B₆Cu₁ significantly raises *T*_*C*_ above room temperature and results in the highest observed -Δ*S*_*M*_^max^ and *RC* in this alloy system [165]. In Fe₉₀-ₓNiₓZr₁₀ ribbons (*x* = 0, 5, 10, 15), increasing the Ni content leads to a systematic rise in *T*_*C*_ from 245 K (*x* = 0) to 403 K (*x* = 15), while maintaining a relatively stable -Δ*S*_*M*_^max^ around 3 J/kg·K under a 4 T field change [166]. These tunable properties suggest that Fe-based ribbons, especially those incorporating Cu or Ni, could be excellent candidates for use in laminate composite structures as magnetic beds in advanced magnetic refrigeration systems.

**Figure 9.**
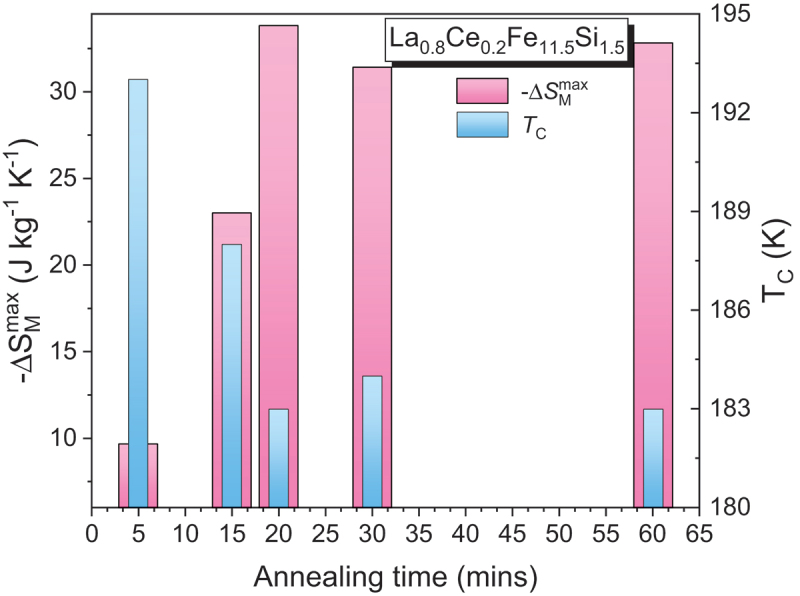
Curie temperature (*T*_*C*_) and maximum magnetic entropy change (-Δ*S*_*M*_^max^) as functions of annealing time for La₀.₈Ce₀.₂Fe₁₁.₅Si₁.₅ alloys ribbons annealed at 1273 K under a field of 1.5 T.

Among intermetallic compounds reported, La(Fe,Si)_13_-based alloys have garnered significant attention for MCEs and magnetic refrigeration due to the relative abundance and low cost of their constituent elements (La, Fe, and Si) compared to Gd-based alternatives [15,167]. These alloys exhibit a strong magneto-structural transition near room temperature in the La(Fe,Si)₁₃ (1:13) phase, which results in a large -Δ*S*_*M*_^max^ (up to 30 J/kg K) and Δ*T*_*ad*_ (up to 12 K) in the temperature range of 270–300 K, making them ideal for household and commercial cooling applications. The Curie temperature of these alloys can be precisely adjusted by modifying the composition (e.g. through hydrogenation or Co substitution), enabling fine tuning of the working temperature range [168,169]. For instance, hydrogenated variants like LaFe₁₁.₆Si₁.₄Hₓ exhibit a shift in the phase transition to higher temperatures, increasing their adaptability for various cooling applications [170]. However, hydrogenation can render these alloys brittle, leading to cracking or powdering during mechanical cycling or active AMR operation. Due to their FOMT nature, La(Fe,Si)₁₃ alloys typically suffer from large thermal and magnetic hysteresis, which leads to energy losses, reduces cooling efficiency, and decreases reversibility during cycling. When compared to their bulk counterparts, as-quenched ribbons of La(Fe,Si)₁₃-based alloys tend to exhibit reduced *T*_*C*_ and -Δ*S*_*M*_^max^ values. Therefore, specialized heat treatments are essential to optimize both the magnetic and magnetocaloric properties of these ribbons [167,171]. Huo *et al*. investigated the formation of the 1:13 phase during rapid solidification by examining the microstructures of the wheel-side and free-side surfaces of melt-spun ribbons [171]. They found that on the free-side, clusters of similarly oriented crystallites formed, with chemical segregation of La, Fe, and Si leading to nanoscale texturing of α-Fe and LaFeSi. In contrast, the wheel-side surface exhibited equiaxed 1:13 grains (∼100–400 nm), with a minor α-Fe phase precipitated in the matrix. Upon annealing, the 1:13 phase grew via the dissolution of the α-Fe phase on the wheel side and a peritectoid reaction from the free side. For longer annealing times, this peritectoid reaction significantly improved the magnetic entropy change under a magnetic field change of 1.5 T, increasing the -Δ*S*_*M*_^max^ from 12 J/kg·K (2 min) to 17 J/kg·K (2 h), and elevated the *T*_*C*_ of the ribbons from 189 K to 201 K. In another case, increasing the annealing time from 10 minutes to 60 minutes for La₀.₈Ce₀.₂Fe₁₁.₅Si₁.₅ ribbons annealed at 1273 K resulted in a substantial increase in -Δ*S*_*M*_^max^ from 9.7 J/kg·K to 32.8 J/kg·K, with a slight reduction in *T*_*C*_ (193 K to 183 K) (see Figure 9).

Additionally, *X*₂Fe₁₇ (*X* = Nd, Y, Pr) ribbons have been shown to exhibit significant -Δ*S*_*M*_^max^ values ranging from 3.7 to 4.8 J/kg·K and *RC* values between 496 and 580 J/kg around room temperature [155,172,173]. Incorporating Nd into Pr₂Fe₁₇ alloys to form Pr₂-ₓNdₓFe₁₇ ribbons (where *x* = 0.5 and 0.7) has resulted in enhanced *T*_*C*_, -Δ*S*_*M*_^max^, and *RC*, with the optimal values observed at *x* = 0.7 [174].

Recently, ribbons of certain high-entropy alloys (HEAs), such as Tm₁₀Ho₂₀Gd₂₀Ni₂₀Al₂₀ and Gd₂₀Dy₂₀Er₂₀Co₂₀Al₂₀, have been explored for use in cryogenic magnetic refrigeration [154,175]. The incorporation of multiple rare-earth and transition metal elements in these alloys leads to a broadened temperature dependence of the magnetic entropy change near their magnetic ordering temperatures. This broadening effect contributes to enhanced *RC* values, while -Δ*S*_*M*_^max^ values are typically reduced, as summarized in Table 3.

### 3.4. Microwires

While Gd can be synthesized in the form of nanoparticles and thin films using chemical or sputtering techniques [7,25,44–46,65,66,97,99–101,125–127], it cannot be readily fabricated into microwires using rapid quenching methods such as melt spinning, in-rotating-water quenching, or glass-coated melt extraction. These techniques typically rely on forming amorphous or metastable phases, which require materials with high glass-forming ability. However, Gd, being a crystalline rare-earth metal, exhibits very poor glass-forming ability and crystallizes rapidly, even under extremely high cooling rates. This rapid crystallization inhibits uniform wire formation. Additionally, Gd is highly reactive, especially at elevated temperatures. During the melting or quenching process, it readily oxidizes to form Gd_2_O_3_, which degrades both its magnetic and structural properties [10]. Gd is also mechanically brittle, making it incompatible with standard wire fabrication methods [10]. These factors collectively make direct fabrication of Gd wires via rapid quenching techniques technically challenging.

To overcome these limitations, Gd has been alloyed with other elements such as Co, Fe, and Al to form compositions like Gd-Co-Al and Gd-Fe-Al [10,31,32]. These alloys possess improved glass-forming ability and can be successfully processed into high-quality microwires using melt-extraction techniques [10]. Numerous Gd-based microwires have been fabricated, and their magnetic and magnetocaloric properties have been widely investigated [7,30–32,35,160,249–262]. These microwires are produced under extremely rapid cooling rates (up to 10^6^ K/s), which results in more homogeneous amorphous structures with fewer inhomogeneities and magnetic clusters than their bulk glass counterparts. This structural uniformity leads to sharper magnetic transitions and enhanced MCEs. For example, Gd₅₅Al₂₀Co₂₅ amorphous microwires exhibit increased -Δ*S*_*M*_^max^ and *RC*, with values of 9.69 J/kg·K and 580 J/kg, respectively, compared to 8.8 J/kg·K and 541 J/kg for their bulk glass equivalents under a 5 T field [249]. Similar improvements are observed in Gd₅₃Al₂₄Co₂₀Zr₃ microwires (10.3 J/kg·K and 733 J/kg) versus bulk samples (9.6 J/kg·K and 690 J/kg) [30].

Notably, most reported MCE data in the literature are obtained using magnetometry on bundles of microwires [30–32,35,160,251–281], rather than single-wire measurements [250]. Comparative studies show that multi-wire samples of Gd₅₃Al₂₄Co₂₀Zr₃ demonstrate superior MCE performance (-Δ*S*_*M*_^max^ of 10.3 J/kg·K and *RC* of 733 J/kg) compared to a single wire (8.8 J/kg·K and 600 J/kg) (Table 4). This enhancement can be attributed to multiple factors, notably averaging effects and magnetostatic interactions. In bundled microwires, variations in diameter, composition, and internal stress among individual wires are effectively averaged out, leading to broader and more uniform magnetic transitions that improve the *RC* [10]. Additionally, the close proximity of wires facilitates dipolar (magnetostatic) interactions, which can amplify the overall magnetization change (Δ*M*) and, consequently, the Δ*S*_*M*_. However, wire – wire interactions can also negatively affect performance through magnetic pinning, depending on spacing, orientation, matrix material, and applied field geometry. Therefore, detailed reporting on the number and arrangement of wires used in measurements is essential for accurate comparison.

**Table 4.** Maximum entropy change, $\left|\Delta S_{M}^{\max}\right|$, Curie temperature, *T*_C_, refrigerant capacity (*RC*), and relative cooling power (*RCP*) for the microwire samples. Values from microwires of other compositions, bulk glasses and Gd are included for comparison.

Microwires	*T*_C_ (K)	µ_0_∆*H* (T)	$\left|\Delta S_{M}^{\max}\right|$ (J/kg K)	*RC* (J/kg)	*RCP* (J/kg)	Ref.
***Gadolinium and its alloys***						
Gd (*B*)	294	5	10.2	410	–	[7]
Gd_55_Co_20_Al_25_ (*B*)	103	5	8.8	541	–	[254]
Gd_55_Al_20_Co_25_ (*MW; A*)	110	5	9.69	580	804	[249]
Gd_55_Co_20_Al_25_ (*MW; A+C*)	100	5	10.1	653	870	[31]
Gd_50_Co_20_Al_30_ (*MW; A+C*)	86	5	10.1	672	896	[31]
Gd_60_Co_20_Al_20_ (*MW; A+C*)	109	5	10.1	681	908	[31]
Gd_55_Co_30_Al_15_ (*MW; A*)	127	5	9.71	573	702	[255]
Gd_60_Co_15_Al_25_ (*MW; A*)	100	5	9.73	732	976	[256]
Gd_60_Co_25_Al_15_ (*R; A*)	125	5	10.1	645	860	[160]
Gd_60_Al_20_Co_20_ (*MW; A+C*)	113	5	10.12	698	936	[251]
Gd_60_Fe_20_Al_20_ (*MW; A*)	202	5	4.8	687	900	[32]
Gd_53_Al_24_Co_20_Zr_3_ *B; A*	95 95	5 3	9.6 6.2	690 340	--	[30]
Gd_53_Al_24_Co_20_Zr_3_ (*MW; A*)	94 94	5 3	10.3 6.9	733 420	--	[30]
Gd_53_Al_24_Co_20_Zr_3_ (*SW; A*)	100	3	5.32	467	555	[257]
Gd_53_Al_24_Co_20_Zr_3_ (*SW; A*)	94 94	5 2	8.8 4.3	600 220	774 296	[250]
Gd_53_Al_24_Co_20_Zr_3_ *SW; C; Annealed at 100* ^*o*^*C*	94 94	5 2	9.5 4.7	687 285	893 348	[250]
Gd_53_Al_24_Co_20_Zr_3_ *SW; C; Annealed at 200* ^*o*^*C*	93 93	5 2	8.0 3.8	629 243	744 307	[250]
Gd_53_Al_24_Co_20_Zr_3_ *SW; C; Annealed at 300* ^*o*^*C*	92 92	5 2	5.1 2.4	396 144	525 184	[250]
Gd_55_Co_25_Ni_20_ (*B*)	78	5	8.0	640	–	[254]
Gd_55_Co_30_Ni_5_Al_10_ (*MW; A*)	140	5	8.91	532	668	[255]
Gd_55_Co_30_Ni_10_Al_5_ (*MW; A*)	158	5	7.68	523	653	[255]
Gd_55_Co_20+x_Ni_10_Al_15-x_ (*MW; A*) *x* = 10 *x* = 5 *x* = 0	158 128 113	5 5 5	7.68 9.00 9.67	546.3 548.9 609.5	681.0 675.0 749.5	[253]
Gd_73.5_Si_13_B_13.5_/GdB_6_ (*MW; A+C*)	106	5	6.4	790	885	[35]
Gd_3_Ni/Gd_65_Ni_35_ (*MW; A+C*)	120	5	9.64	742	–	[252]
Gd_50_-(Co_69.25_Fe_4.25_Si_13_B_13.5_)_50_ (*MW; A*)	170	5	6.56	625	826	[258]
Gd_59.4_Al_19.8_Co_19.8_Fe_1_ (*MW; A*)	113	5	10.33	748	1006	[259]
(Gd_60_Al_20_Co_20_)_99_Ni_1_ (*MW; A+C*)	111	5	10.98	725.49	970.89	[251]
(Gd_60_Al_20_Co_20_)_97_Ni_3_ (*MW; A+C*)	109	5	11.06	746.84	1000.50	[251]
(Gd_60_Al_20_Co_20_)_95_Ni_5_ (*MW; A+C*)	109	5	11.57	834.14	1138.16	[251]
(Gd_60_Al_20_Co_20_)_93_Ni_7_ (*MW; A+C*)	108	5	10.77	733.48	977.65	[251]
Gd_36_Tb_20_Co_20_Al_24_ (*MW; A*)	91	5	12.36	731	948	[260]
Gd_36_Tb_20_Co_20_Al_24_ (*MW; A+C*)	81	5	8.8	500	625	[260]
(Gd_36_Tb_20_Co_20_Al_24_)_99_Fe_1_ (*MW; A+C*)	94	5	8.5	510	635	[260]
(Gd_36_Tb_20_Co_20_Al_24_)_98_Fe_2_ (*MW; A+C*)	100	5	8.0	515	660	[260]
(Gd_36_Tb_20_Co_20_Al_24_)_97_Fe_3_ (*MW; A+C*)	108	5	7.6	520	680	[260]
Gd_19_Tb_19_Er_18_Fe_19_Al_25_ (*MW; A+C*)	97	5	5.94	569	733	[261]
Gd_36_Tb_20_Co_20_Al_24_ (*MW; A+C*)	82	5	9	518	657	[262]
***Intermetallics compounds***						
HoErCo (*MW; A*)	16	5	15	527	600	[268]
HoErFe (*MW; A+C*)	44	5	9.5	450	588	[269]
DyHoCo (*MW; A*)	35	5	11.2	417	530	[270]
Mn_x_Fe_2-x_P_0.5_Si_0.5_ (*M; C*) *x* = 0.7 *x* = 0.8 *x* = 0.9 *x* = 1.0 *x* = 1.1 *x* = 1.2	> 400 351 298.5 263 235.5 190	5 5 5 5 5 5	-12 18.3 15.8 10.8 1.2	-293.7 331.1 300 280.9 288.4	--	[264]
MnFe_x_P_0.5_Si_0.5_ (*M; C*) *x* = 0.9 *x* = 0.95 *x* = 1.0 *x* = 1.05	311 281 263 245.5	5 5 5 5	10.7 10.3 15.8 14.5	295.8 286.2 300.0 283.9	293.7 275.3 257.1 243.1	[266] (*)
(MnFe)_*x*_(P_0.5_Si_0.5_) (*W*, *C*) *x* = 1.85 *x* = 1.90 *x* = 1.95 *x* = 2.00	355 370 340 263	5 5 5 5	16.3 26.0 19.4 15.8	~308 ~367 ~325 ~295		[265] (*)
Mn_1.3_Fe_0.6_P_0.5_Si_0.5_, as-cast (*M; C*) Mn_1.3_Fe_0.6_P_0.5_Si_0.5_, annealed (*M; C*)	138 145	2 5 2 5	1.9 4.6 5.1 10.5	160-178 440	--	[271]
Mn_1.26_Fe_0.60_P_0.48_Si_0.52_ (*MW; C*)	141	5	4.64	–	–	[271]
Dy_36_Tb_20_Co_20_Al_24_ (*MW; A+C*)	42	5	8.2	301	414	[262]
Ho_36_Tb_20_Co_20_Al_24_ (*MW; A+C*)	42	5	10.3	372	474	[262]
LaFe_11.6_Si_1.4_ (*MW; A*)	195	2	9.0	–	45	[272]
***Heusler alloys***						
Ni_2_MnGa (*GCW; C; Annealed)*	315	3	0.7	–	–	[273]
Ni_50.5_Mn_29.5_Ga_20_ (*MW; C*)	368	5	18.5	63	–	[274] (*)
Ni_50.6_Mn_28_Ga_21.4_ (*MW; C*)	340–370	5	5.2	240	–	[274]
Ni_48_Mn_26_Ga_19.5_Fe_6.5_ (*MW; C*)	361	5	4.7	–	–	[275]
Ni_49.4_Mn_26.1_Ga_20.8_Cu_3.7_ (*MW; C*)	359	5	8.3	78	–	[276] (*)
Ni_45.6_Fe_3.6_Mn_38.4_Sn_12.4_ (*MW; A+C*)	270 300	5 5	15.2 4.3	146 175	182 215	[277] (*)
Ni_48_Mn_25.6_Ga_19.4_Fe_6.5_ (*MW; A*)	361	5	4.7	–	18	[278]
Ni_48.5_Mn_26_Ga_19.5_Fe_6.5_ (*MW; C*)	391	5	2.91	–	–	[275]
Ni_44.9_Fe_4.3_Mn_38.3_Sn_12.5_ (*MW; C; Annealed*)	299 FOMT	5 5	3.7 6.9	~233 78	--	[279] (*)
Ni_45_Mn_37_In_13_Co_5_ *(GCW; C; Annealed*)	315	5	0.5	–	–	[280]
Ni_50.95_Mn_25.45_Ga_23.6_ (*GCW; C; Annealed*)	315	3	0.7	–	–	[281]

SW: Single wire; MW: Multiple wires; B: Bulk; R: Ribbon; GCW: Glass-coated wires. A: Amorphous; C: Crystalline; M: Microwires; (*) represents FOMT materials.

#### 3.4.1. Annealing and structural optimization

**Figure 10.**
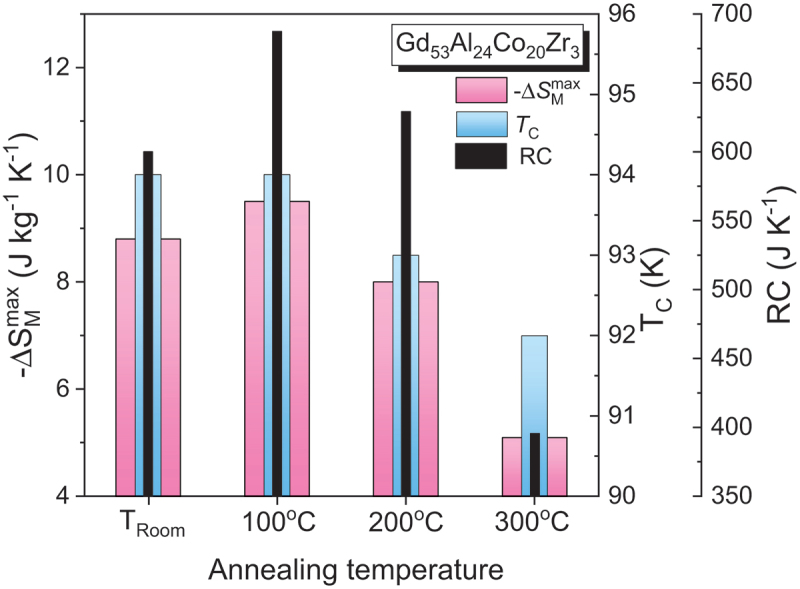
Curie temperature (*T*_C_), maximum magnetic entropy change (−Δ*S*_M_^max^), and refrigerant capacity (*RC*) as functions of annealing temperature for Gd₅₃Al₂₄Co₂₀Zr₃ alloys wires, including as-spun amorphous ribbon, crystallized ribbons annealed at various temperatures, and bulk sample under a magnetic field change of 5 T.

Amorphous microwires often undergo thermal annealing to further improve their magnetic and magnetocaloric properties [31,250,251]. Annealing promotes structural relaxation and controlled nanocrystallization, optimizing the microstructure for magnetic ordering and energy conversion. As-quenched wires contain high levels of defects and internal stress; low-temperature, short-duration annealing relieves these stresses and facilitates atomic rearrangement while retaining the amorphous phase. For instance, Gd₅₃Al₂₄Co₂₀Zr₃ microwires annealed at 100°C exhibit significant improvements, achieving a -Δ*S*_*M*_^max^ of 9.5 J/kg·K and *RC* of 689 J/kg, as shown in Figure 10 [250]. This *RC* is 35%–91% higher than that of bulk samples. The annealed wires show formation of nanocrystallites (5–10 nm in size) embedded in the amorphous matrix, leading to lattice distortions that alter magnetic properties and increase mechanical strength (up to 1845 MPa at 100 °C). This dual-phase (amorphous + nanocrystalline) structure is found desirable for enhancing both magnetocaloric and mechanical responses.

#### 3.4.2. Compositional engineering and melt-extraction control

**Figure 11.**
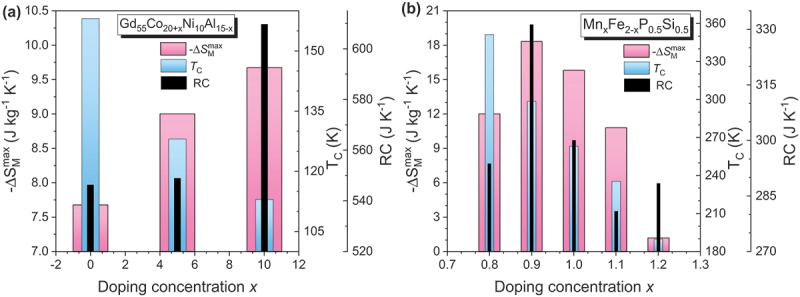
Curie temperature (*T*_C_), maximum magnetic entropy change (−Δ*S*_M_^max^), and refrigerant capacity (*RC*) as functions of Co doping concentration (*x*) in (a) Gd₅₅Co₂₀+xNi₁₀Al₁₅-_x_ wires (*x* = 10, 5, and 0) and (b) Mn_x_Fe_2−x_P_0.5_Si_0.5_ wires for *x* = 0.7 to 1.2 under a field change of 5 T.

The nanocrystalline/amorphous structure can also be tailored during melt-extraction itself. In Gd_(50 + 5x)_Al_(30 − 5x)_Co₂₀ (*x* = 0, 1, 2) microwires, about 20% of uniformly distributed ~10 nm nanocrystallites embedded in the amorphous matrix enhanced magnetocaloric response [31]. These microwires displayed large values of -Δ*S*_*M*_^max^ (~9.7 J/kg·K), Δ*T*_*ad*_ (~5.2 K), and *RC* (~654 J/kg) under a 5 T field. Gd enrichment significantly adjusts the *T*_*C*_ while preserving high Δ*S*_*M*_ and *RC* values. This structural configuration also broadens the operating temperature span of magnetic beds, which is critical for energy-efficient magnetic refrigeration. Additionally, novel composite microwires with embedded antiferromagnetic nanocrystals, such as GdB₆ in an amorphous ferromagnetic Gd₇₃.₅Si₁₃B₁₃.₅ matrix, showed promising MCE behavior (-Δ*S*_*M*_^max^ ≈6.4 J/kg·K, *RC* ≈890 J/kg) over wide temperature intervals (~130 K) [35]. Similar effects were reported in Gd₃Ni/Gd₆₅Ni₃₅ composite microwires [252]. By tailoring magnetic interactions, including RKKY ferromagnetic (Gd–Gd) and antiferromagnetic (Gd–Co, Gd–Ni) couplings, researchers have demonstrated the potential to fine-tune *T*_*C*_ while maintaining high *RC* in Gd₅₅Co₂+ₓNi₁₀Al₁₅-ₓ (*x* = 0, 5, 10) microwires, as can be seen in Figure 11(a) [253].

#### 3.4.3. Toward tunable magnetic beds and room-temperature MCE

An important advantage of Gd-based alloy microwires is their tunable *T*_*C*_ through compositional design, enabling the selection of wires with staggered *T*_*C*_ and high Δ*S*_*M*_. This allows for the construction of engineered magnetic beds with laminate structures, achieving a table-like MCE response – ideal for Ericsson-cycle magnetic refrigeration systems [263]. However, Gd-based microwires are mostly limited to cryogenic and sub-room-temperature ranges (90–150 K). To enable ambient temperature applications, alternative systems are under investigation. Luo *et al*. reported a tunable giant MCE around room temperature in Mn_x_Fe₂-ₓP₀.₅Si₀.₅ (0.7 ≤ *x* ≤1.2) microwires produced via melt-extraction and thermal treatment [264]. By adjusting Mn/Fe ratios, *T*_*C*_ was varied from 190 to 351 K, and a large -Δ*S*_*M*_^max^ of 18.3 J/kg·K at 300 K was achieved for *x* = 0.9 (see Figure 11(b)). After accounting for magnetic hysteresis loss due to the FOMT nature, the *RC* was ~285 J/kg. Ongoing work focuses on reducing magnetic losses while maintaining high Δ*S*_*M*_. For instance, controlling the metal-to-nonmetal ratio (M/NM = *x*:1) in (MnFe)ₓ(P₀.₅Si₀.₅) (*x* = 1.85–2.0) microwires can reduce thermal and magnetic hysteresis by up to 40%, with -Δ*S*_*M*_^max^ and *RC* reaching optimal values at *x* = 1.90 (-Δ*S*_*M*_^max^ ~26.0 J/kg·K; *RC* ~367.4 J/kg; *T*_*C*_ ~370 K) [265]. The effect of Fe content on the microstructure, magnetic, and magnetocaloric properties of MnFe_x_P₀.₅Si₀.₅ (0.9 ≤ *x* ≤1.05) microwires has also been investigated [266]. As the Fe content increases, the system undergoes a transition from a FOMT for *x* = 1.00 and 1.05 to a SOMT for *x* = 0.90 and 0.95, leading to reduced magnetic losses but also a decrease in both the -Δ*S*_*M*_^max^ and *RC*.

Heusler alloy microwires (e.g. Ni₅₀.₅Mn₂₉.₅Ga₂₀ and Ni₄₅.₆Fe₃.₆Mn₃₈.₄Sn₁₂.₄) have also shown significant -Δ*S*_*M*_^max^ (up to 18.5 J/kg·K) in the sub-room and room temperature regions, though their *RC* values (60–230 J/kg) remain much lower than those of GdCo- or MnFe-based microwires [273–281]. Similar to their ribbon and thin film counterparts, Heusler alloy microwires exhibit SOMT ferromagnetic ordering at or above room temperature, followed by a FOMT at lower temperatures. Typically, larger Δ*S*_*M*_ values are observed near the FOMT, though within a relatively narrow temperature span. In contrast, Δ*S*_*M*_ values associated with the SOMT are smaller but distributed over a broader temperature range. As a result, certain Heusler microwire systems demonstrate enhanced *RC* around the SOMT. Through careful compositional tuning, hysteretic magnetic losses, particularly those associated with the FOMT, can be minimized, enabling improvements in *RC* while maintaining strong MCE performance. Additionally, targeted thermal treatments are critical for optimizing the microstructure and enhancing the overall MCE properties of Heusler alloy microwires.

Recently, the MCE in high-entropy magnetic materials has garnered increasing attention for magnetic refrigeration applications, primarily due to their excellent mechanical and magnetic properties [154,175,267]. High-entropy alloy microwires typically exhibit reduced Δ*S*_*M*_ (*T*) peaks but over significantly broader temperature ranges compared to conventional magnetocaloric materials. Notably, Yin *et al*. demonstrated that the magnetocaloric properties of high-entropy alloy microwires with the composition (Gd₃₆Tb₂₀Co₂₀Al₂₄)₁₀₀-ₓFeₓ can be significantly improved through current annealing of their as-cast amorphous counterparts [267]. This treatment induces the controlled precipitation of nanocrystals within the amorphous matrix, creating phase compositional heterogeneity along the microwires. The resulting microstructure broadens the temperature range of the Δ*S*_*M*_ and thereby enhances the *RC* in the annealed samples. While current annealing can enhance both MCE and *RC*, it is equally important to maintain the exceptional mechanical integrity characteristic of these high-entropy systems.

For cryogenic applications, microwires of rare-earth-based compositions such as HoErCo, HoErFe, DyHoCo, and Dy₃₆Tb₂₀Co₂₀Al₂₄ show large -Δ*S*_*M*_^max^ values (~10 J/kg·K), making them attractive candidates for cryogenic magnetic cooling applications [268–270]. However, the mechanical properties of these systems remain largely unexplored.

## 4. Material candidates for energy-efficient magnetic refrigeration

**Figure 12.**
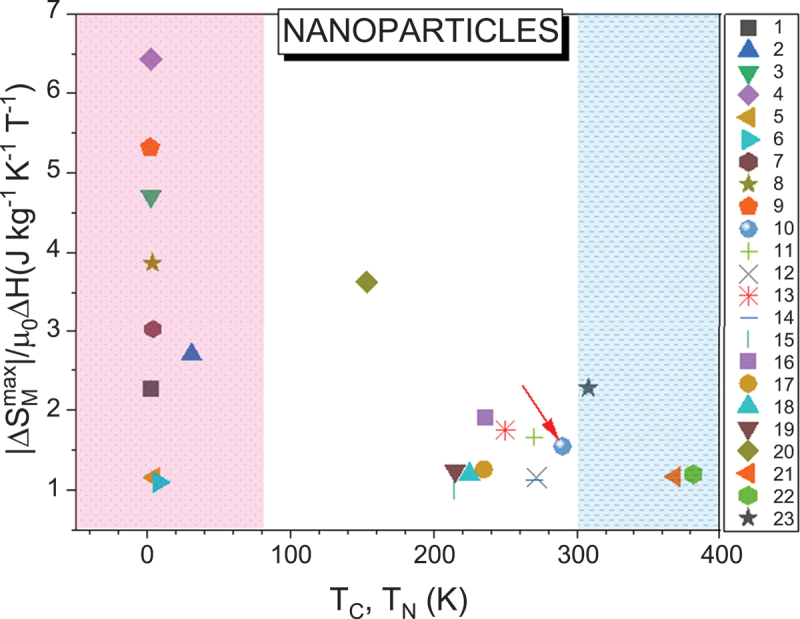
Performance coefficients (|Δ*S*_*M*_^*max*^*|*/μ₀Δ*H*_max_) of magnetocaloric nanoparticles evaluated at their respective Curie (*T*_*C*_) or Néel (*T*_*N*_) temperatures across three cooling temperature regimes: low (*T* < 80 K), intermediate (80 K < *T* < 300 K), and high (*T* > 300 K). *low-temperature range*: 1-MnPS_3_ [91]; 2-GdNi_5_ [66]; 3-GdVO_4_-30 nm [85]; 4-GdVO_4_-300 nm [85]; 5-Gd_3_Fe_5_O_12_ [58]; 6-Tb_2_O_3_ [61]; 7-Dy_2_O_3_ [61]; 8-Gd_2_O_3_ [61]; 9-Ho_2_O_3_ [61]; *intermediate-temperature range*: 10-Gd [45]; 11-La_0.6_Ca_0.4_MnO_3_-223 nm [47]; 12-La_0.6_Ca_0.4_MnO_3_-122 nm [47]; 13-La_0.67_Ca_0.33_MnO_3_ [70]; 14-La_0.7_Ca_0.3_MnO_3_ [71]; 15-La_0.8_Ca_0.2_MnO_3_-28 nm [53]; 16-La_0.8_Ca_0.2_MnO_3_-43 nm [53]; 17-Pr_0.7_Sr_0.3_MnO_3_ [78]; 18-Pr_0.65_(Ca_0.7_Sr_0.3_)_0.35_MnO_3_ [81]; 19-La_0.35_Pr_0.275_Ca_0.375_MnO_3_ [63]; 20-DyCrTiO_3_ [60]; *high-temperature range*: 21-La_0.67_Sr_0.33_MnO_3_ [50]; 22-MnFeP_0.45_Si_0.55_ [92]; 23-La_0.7_Ca_0.2_Sr_0.1_MnO_3_ [84].

**Figure 13.**
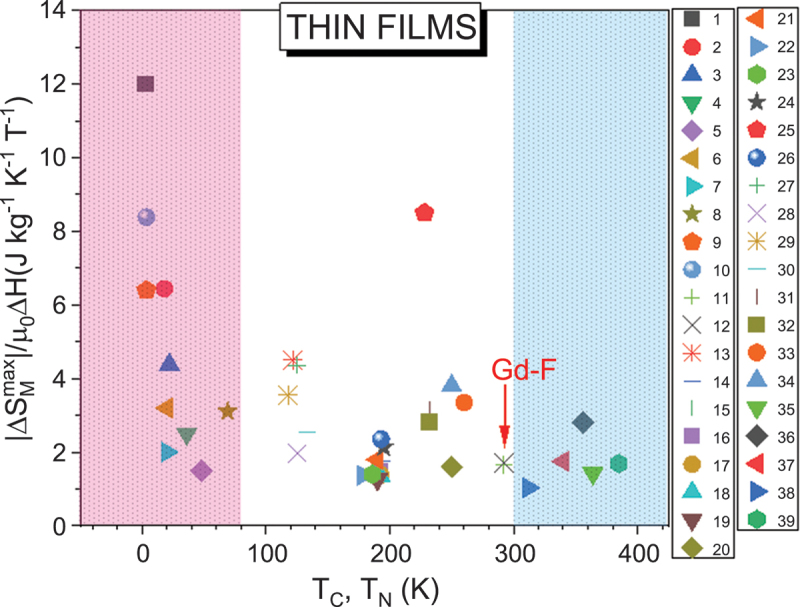
Performance coefficients (|Δ*S*_*M*_^*max*^*|*/μ₀Δ*H*_max_) of magnetocaloric thin films evaluated at their respective Curie (*T*_*C*_) or Néel (*T*_*N*_) temperatures across three cooling temperature regimes: low (*T* < 80 K), intermediate (80 K < *T* < 300 K), and high (*T* > 300 K). *low-temperature range*: 1-EuTiO_3_ [113]; 2-CrF_3_ [111]; 3-CrCl_3_ [111]; 4-CrBr_3_ [111]; 5-CrI_3_ [111]; 6-Fe_3_[Cr(CN)_6_]_2_⋅zH_2_O at 1 T [145]; 7-Fe_3_[Cr(CN)_6_]_2_⋅zH_2_O at 5 T [145]; 8-EuO_1_ [110]; 9-GdCoO_3_/LAO at 2 T [115]; 10-GdCoO_3_/LAO at 7 T [115]; *intermediate-temperature range*: 11-Gd (F, *t* = 17μm) [97]; 12-Gd (F, *t* = 30 nm) annealed at 450 K [25]; 13-GdSi_2_ [125]; 14-Gd_5_Si_1.3_Ge_2.7_ [101]; 15-Gd_5_Si_1.3_Ge_2.7_ (thermal cycling, 50 cycles) [101]; 16-Gd_5_Si_1.3_Ge_2.7_ (thermal cycling, 200 cycles) [101]; 17-Gd_5_Si_1.3_Ge_2.7_ (thermal cycling, 250 cycles) [101]; 18-Gd_5_Si_1.3_Ge_2.7_ (thermal cycling, 450 cycles) [101]; 19-Gd_60_Co_40_ [99]; 20-Ni_51.6_Mn_32.9_Sn_15.5_ [131]; 21-La_0.7_Ca_0.3_MnO (extrinsic) [103]; 22-La_0.8_Ca_0.2_MnO_3_/STO (tensile strain, *t* = 25 nm) [106]; 23-La_0.8_Ca_0.2_MnO_3_/STO (tensile strain, *t* = 50 nm) [106]; 24-La_0.8_Ca_0.2_MnO_3_/STO (tensile strain, *t* = 75 nm) [106]; 25-La_2/3_Ca_1/3_MnO_3_ [133]; 26-Pr_0.7_Sr_0.3_MnO_3_/PSMO-7 [135]; 27-Gd_2_NiMnO_6_ (in-plane) [120]; 28-Gd_2_NiMnO_6_ (out of plane) [120]; 29-EuO_0.975_ [110]; 30-EuO_0.91_ [110]; 31-epitaxial Tb (H//a axis, in-plane) [121]; 32-epitaxial Tb (H//b axis, in-plane) [121]; 33-Ni_80_Fe_20_/Ni_67_Cu_33_/Co_90_Fe_10_/Mn_80_Ir_20_ (Spacer = Ni_67_Cu_33_, *t* = 7 nm) [119]; 34-Ni_80_Fe_20_/Ni_67_Cu_33_/Co_90_Fe_10_/Mn_80_Ir_20_ (Spacer = Ni_67_Cu_33_, *t* = 10 nm) [119]; *high-temperature range*: 35-Ni_53.5_Mn_23.8_Ga_22.7_ [128]; 36-Ni_51_Mn_29_Ga_20_ [129]; 37-Ni_43_Mn_32_Ga_20_Co_5_ [132]; 38-La_0.67_Sr_0.33_MnO_3_ [105]; 39-CrO_2_/TiO_2_ [142].

**Figure 14.**
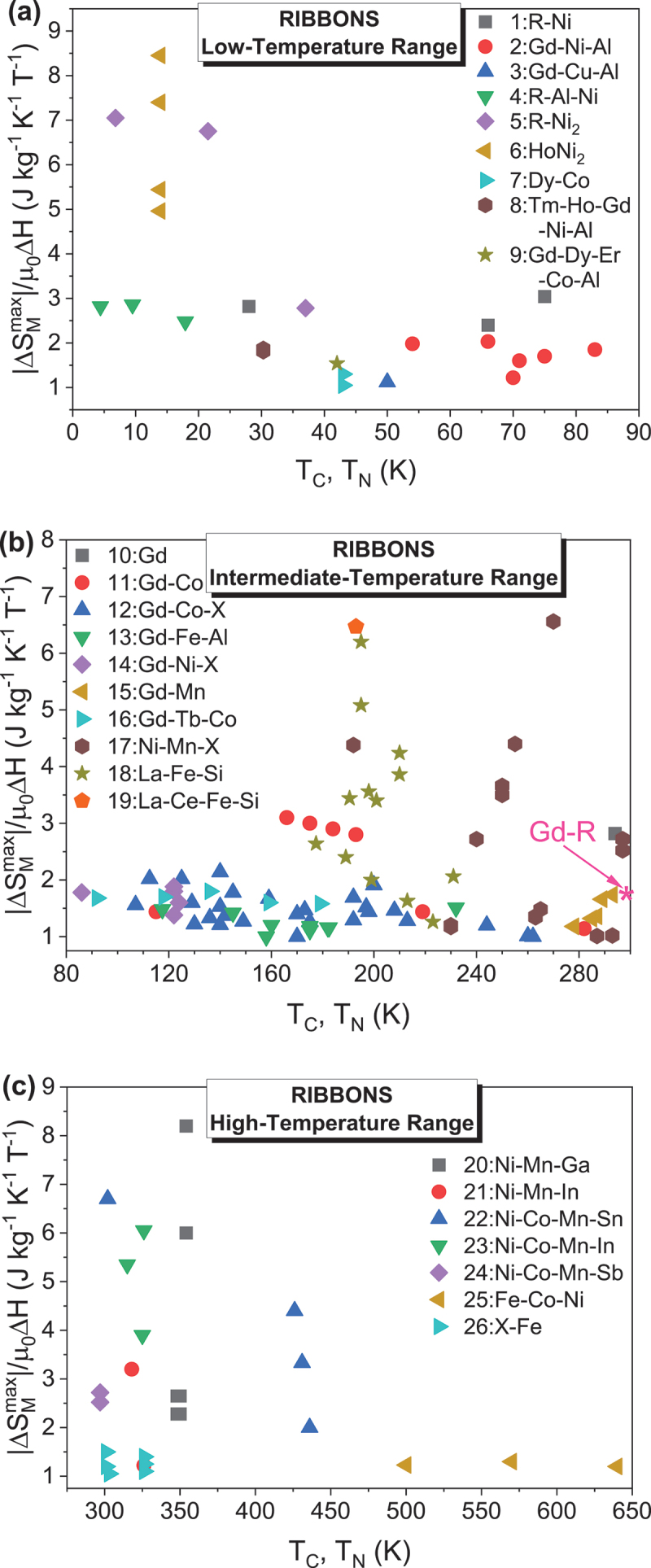
Performance coefficients (|Δ*S*_*M*_^*max*^*|*/μ₀Δ*H*_max_) of magnetocaloric ribbons evaluated at their respective Curie (*T*_*C*_) or Néel (*T*_*N*_) temperatures across three temperature cooling regimes: (a) low (*T* < 80 K), (b) intermediate (80 K < *T* < 300 K), and (c) high (*T* > 300 K). *low-temperature range*: 1: R-Ni [188]; 2: Gd-Ni-al [163,190-]1192]; 3: Gd-Cu-al [195]; 4: R-Al-Ni [205]; 5: R-Ni_2_ [206–208]; 6: HoNi_2_ [209]; 7: Dy-Co [210]; 8: Tm-Ho-Gd-Ni-al [175]; 9: Gd-Dy-Er-Co-al [154]; *intermediate-temperature range*: 10: Gd [157]; 11: Gd-Co [157,158,177]; 12: Gd-Co-X [160,161;179–185]; 13: Gd-Fe-al [162;186–188]; 14: Gd-Ni-X [159,163,188–191]; 15: Gd-Mn [157]; 16: Gd-Tb-Co [194]; 17: Ni-Mn-X [19,212–214,216–227]; 18: La-Fe-Si [171,245,246]; 19: La-Ce-Fe-Si [247]; *high-temperature range*: 20: Ni-Mn-Ga [214,218]; 21: Ni-Mn-in [219,223]; 22: Ni-Co-Mn-Sn [222,226]; 23: Ni-Co-Mn-in [224-2226]; 24: Ni-Co-Mn-Sb [221]; 25: Fe-Co-Ni [240]; 26: X-Fe [155,172,244].

**Figure 15.**
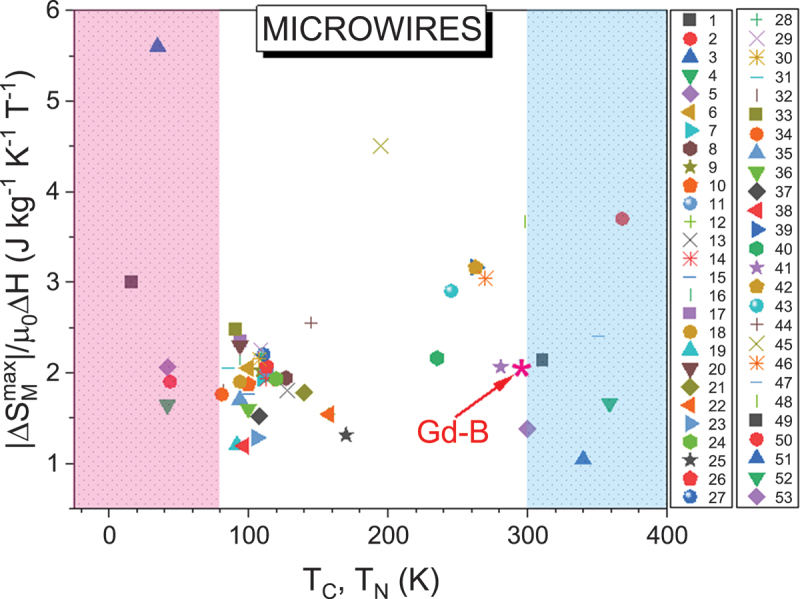
Performance coefficients (-Δ*S*_*M*_^*max*^/μ₀Δ*H*_max_) of magnetocaloric microwires evaluated at their respective Curie (*T*_*C*_) or Néel (*T*_*N*_) temperatures across three temperature cooling regimes: low (*T* < 80 K), intermediate (80 K < *T* < 300 K), and high (*T* > 300 K). *low-temperature range*: 1-HoErCo [268]; 2-HoErFe [269]; 3-DyHoCo [270]; 4-Dy_36_Tb_20_Co_20_Al_24_ [262]; 5-Ho_36_Tb_20_Co_20_Al_24_ [262]; *intermediate-temperature range*: 6-Gd_55_Co_20_Al_25_ [31]; 7-Gd_55_Co_30_Al_15_ [255]; 8-Gd_55_Co_25_Al_20_ [249]; 9-Gd_60_Al_20_Co_20_ [31]; 10-Gd_60_Co_15_Al_25_ [256]; 11-Gd_60_Al_20_Co_20_ [251]; 12-Gd_55_Co_20+x_Ni_10_Al_15-x_ (x = 10) [253]; 13-Gd_55_Co_20+x_Ni_10_Al_15-x_ (*x* = 5) [253]; 14-Gd_55_Co_20+x_Ni_10_Al_15-x_ (*x* = 0) [253]; 15-Gd_53_Al_24_Co_20_Zr_3_ (SW) [257]; 16-Gd_53_Al_24_Co_20_Zr_3_ (SW) [250]; 17-Gd_53_Al_24_Co_20_Zr_3_ (SW, annealed at 100 °C) [250]; 18-Gd_53_Al_24_Co_20_Zr_3_ (SW, annealed at 200 °C) [250]; 19-Gd_53_Al_24_Co_20_Zr_3_ (SW, annealed at 300 °C) [250]; 20-Gd_53_Al_24_Co_20_Zr_3_ (MW) [30]; 21-Gd_55_Co_30_Ni_5_Al_10_ [255]; 22-Gd_55_Co_30_Ni_10_Al_5_ [255]; 23-Gd_73.5_Si_13_B_13.5_/GdB_6_ [35]; 24-Gd_3_Ni/Gd_65_Ni_35_ [252]; 25-Gd_50-_(Co_69.25_Fe_4.25_Si_13_B_13.5_)_50_ [258]; 26-Gd_59.4_Al_19.8_Co_19.8_Fe_1_ [259]; 27-(Gd_60_Al_20_Co_20_)_99_Ni_1_ [251]; 28-(Gd_60_Al_20_Co_20_)_97_Ni_3_ [251]; 29-(Gd_60_Al_20_Co_20_)_95_Ni_5_ [251]; 30-(Gd_60_Al_20_Co_20_)_93_Ni_7_ [251]; 31-Gd_50_Co_20_Al_30_ [31]; 32-Gd_36_Tb_20_Co_20_Al_24_ (A+C) [27]; 33-Gd_36_Tb_20_Co_20_Al_24_ (A) [260]; 34-Gd_36_Tb_20_Co_20_Al_24_ (A+C) [260]; 35-(Gd_36_Tb_20_Co_20_Al_24_)_99_Fe_1_ [260]; 36-(Gd_36_Tb_20_Co_20_Al_24_)_98_Fe_2_ [260]; 37-(Gd_36_Tb_20_Co_20_Al_24_)_97_Fe_3_ [260]; 38-Gd_19_Tb_19_Er_18_Fe_19_Al_25_ [261]; 39-Mn_x_Fe_2-x_P_0.5_Si_0.5_ (*x* = 1) [264]; 40-Mn_x_Fe_2-x_P_0.5_Si_0.5_ (*x* = 1.1) [264]; 41-MnFe_x_P_0.5_Si_0.5_ (*x* = 0.95) [266]; 42-MnFe_x_P_0.5_Si_0.5_ (*x* = 1) [266]; 43-MnFe_x_P_0.5_Si_0.5_ (*x* = 1.05) [266]; 44-Mn_1.3_Fe_0.6_P_0.5_Si_0.5_, annealed [271]; 45-LaFe_11.6_Si_1.4_ [272]; 46-Ni_45.6_Fe_3.6_Mn_38.4_Sn_12.4_ [277]; *high-temperature range*: 47-Mn_x_Fe_2-x_P_0.5_Si_0.5_ (x = 0.8) [264]; 48-Mn_x_Fe_2-x_P_0.5_Si_0.5_ (*x* = 0.9) [264]; 49-MnFe_x_P_0.5_Si_0.5_ (*x* = 0.9) [266]; 50-Ni_50.5_Mn_29.5_Ga_20_ [274]; 51-Ni_50.6_Mn_28_Ga_21.4_ [274]; 52-Ni_49.4_Mn_26.1_Ga_20.8_Cu_3.7_ [276]; 53-Ni_44.9_Fe_4.3_Mn_38.3_Sn_12.5_ [279].

Based on a comprehensive analysis of the magnetocaloric properties across various material forms, including nanoparticles, thin films, ribbons, and microwires, we propose several promising candidates for active magnetic cooling applications, categorized by temperature range: cryogenic (*T* < 80 K), intermediate (80 K < *T* < 300 K), and high temperature (*T* > 300 K). These candidates are highlighted in Figures 12–15, as well as summarized in Table 5.

**Table 5.** Material candidates for energy-efficient magnetic refrigeration applications in the three cooling temperature regimes.

Magnetic Cooling Applications		
Low-Temperature Range (Cryogenic Cooling)	Intermediate-Temperature Range	High-Temperature Range
*T* < 80K	80 K < *T* < 300 K	*T* > 300K
**Applications**: Liquefaction of hydrogen and helium Cryogenics for space technology, superconducting magnets, quantum devices, and sensors	**Applications**: Near-room-temperature cooling Biomedical devices (e.g. magnetic hyperthermia) Electronic component cooling	**Applications**: Industrial waste heat recovery Thermomagnetic energy conversion Magnetic hyperthermia
**Material candidates**: Oxide nanoparticles (e.g. GdVO_4_, Gd_2_O_3_, Ho_2_O_3_, Dy_2_O_3_, Gd₃Ga₅O₁₂) Intermetallic alloy nanoparticles (e.g. MnFeP_0.45_Si_0.05_, Ni_95_Cr_5_, MnPS_3_, GdNi_5_) Oxide films (e.g. GdCoO_3_, EuTiO₃) MnF_2_/FM films Rare-earth based ribbons (e.g. Gd-Ni-Al, *R*-Ni_2_, *R*-Al-Ni) Rare-earth-based microwires (e.g. DyHoCo, HoErCo)	**Material candidates**: Gd nanoparticles, films and ribbons — *T*_*C*_ ~294 K Gd-based films (GdSi_2_, Gd_5_Si_1.3_Ge_2.7_) Oxide films (e.g. EuO, Gd_2_NiMnO_6_, La₀.₇Ca₀.₃MnO₃) Fe-Rh-Pd films Gd-based ribbons (Gd-Co, Gd-Mn, Gd-Co-*X*) La-Fe-Si-based ribbons and microwires Heusler alloy ribbons and microwires (Ni-Mn-*X*)	**Material candidates**: Manganite nanoparticles and films (e.g. La_0.67_Sr_0.33_MnO_3_, La_0.7_Ca_0.1_Sr_0.2_MnO_3_) CrO_2_/TiO_2_ films FeRh and FeRh/BaTiO_3_ films Heusler alloy ribbons and microwires (e.g. Ni-Mn-Gd, Ni-Mn-In, Ni-Co-Mn-In, Ni-Co-Mn-Sn, Ni-Mn-Ga-Cu) *X*-Fe alloy ribbons Mn-Fe-P-Si microwires

Since Δ*S*_*M*_ values are often reported under varying experimental conditions, such as different magnetic field strengths and measurement protocols, it is not straightforward to directly compare the performance of magnetocaloric materials across different studies. To address this, we define a performance coefficient as the ratio of the maximum magnetic entropy change (-Δ*S*_*M*_^*max*^) to the corresponding maximum applied magnetic field change (μ₀Δ*H*_max_). This normalized metric provides a more consistent basis for evaluating the effectiveness of magnetocaloric materials. A performance coefficient greater than one is considered indicative of a promising candidate for magnetic refrigeration. Using this criterion, we highlight a selection of high-potential magnetocaloric materials in various reduced-dimensional forms, including nanoparticles (Figure 12), thin films (Figure 13), ribbons (Figure 14(a-c)), and microwires (Figure 15).

As shown in Figure 12, the majority of magnetocaloric nanoparticle candidates are oxides. Among them, GdVO₄ nanoparticles exhibit the highest performance coefficient in the low-temperature range (*T* < 80 K), while DyCrTiO₃ nanoparticles lead in the intermediate temperature range (80 K < *T* < 300 K). In the high-temperature range (*T* > 300 K), La₀.₇Ca₀.₂Sr₀.₁MnO₃ nanoparticles demonstrate the greatest performance coefficient. Although certain manganite oxide nanoparticles exhibit notable magnetic entropy changes, their inherently high heat capacities often lead to low or moderate adiabatic temperature changes, which can limit their overall cooling efficiency.

In the case of magnetocaloric thin films, various candidate materials are distributed across the three major cooling temperature regimes, as illustrated in Figure 13. In the low-temperature range (*T* < 80 K), EuTiO_3_ exhibits the highest performance coefficient. Within the intermediate temperature range (80 K < *T* < 300 K), GdCoO_3_ shows the strongest performance. At high temperatures (*T* > 300 K), Ni_51_Mn_29_Gd_20_ demonstrates the highest performance coefficient among the thin film candidates. However, the performance coefficient of the Ni₅₁Mn₂₉Gd₂₀ thin film is relatively low compared to other magnetocaloric candidates and requires enhancement to enable its use in AMR. Additionally, the adiabatic temperature change – an even more critical parameter for evaluating magnetocaloric materials – remains largely unexplored in these thin-film systems.

As illustrated in Figure 14, a wide range of ribbon-based magnetocaloric materials are available across the three primary cooling temperature regimes. In the low-temperature range (*T* < 80 K), as shown in Figure 14(a), rare-earth-based ribbons are the leading candidates. In the intermediate temperature range (80 K < *T* < 300 K), Gd and Gd-based alloy ribbons (GdCo, Gd-Co-*X*, Gd-Fe-*X*) dominate (Figure 14(b)). At high temperatures (*T* > 300 K), Heusler alloy ribbons (e.g. Ni-Mn-Ga, Ni-Co-Mn-Sn, Ni-Co-Mn-In) emerge as the principal candidates (Figure 14(c)).

Similar to magnetocaloric ribbons, rare-earth-based microwires (e.g. DyHoCo) are the leading candidates in the low-temperature range (*T* < 80K). In the intermediate temperature range (80 K < *T* < 300 K), Gd alloy-based microwires (e.g. Gd_60_Al_20_Co_20_, Gd_36_Tb_20_Co_20_Al_24_) are the principal candidates. At high temperatures (*T* > 300K), Mn – Fe – P – Si and Heusler alloy-based microwires (e.g. Ni_45.6_Fe_3.6_Mn_38.4_Sn_12.4_) dominate. Although several Heusler alloy microwires exhibit a large magnetic entropy change and a high-performance coefficient, the Δ*S*_*M*_(*T*) is confined to a narrow temperature range, leading to a moderate *RC*, which may limit their suitability for practical cooling applications.

## 5. Challenges and opportunities

Despite their scientific promise, low-dimensional magnetocaloric materials face several key challenges that limit their implementation in active cooling systems. Below, we outline the primary hurdles associated with nanoparticles, thin films, ribbons, and microwires.

Magnetocaloric nanoparticles, while promising for cryogenic and localized cooling, face significant application barriers related to magnetic field requirements, thermal integration, stability, scalability, and device engineering. Ferromagnetic nanoparticles often exhibit suppressed *T*_*C*_ and reduced Δ*S*_*M*_, although refrigerant capacity (*RC* or *RCP*) may improve due to entropy broadening. The diminished Δ*S*_*M*_ reduces the cooling power per cycle, particularly under moderate magnetic fields. Antiferromagnetic nanoparticles may show large Δ*S*_*M*_ but typically require high magnetic fields (3–7 T), necessitating superconducting magnets or bulky setups – hindering the development of compact and energy-efficient devices. Thermally, nanoparticles have low intrinsic conductivity and high interfacial resistance when embedded in fluids or solids, making efficient heat transfer to/from the load difficult. They are also prone to agglomeration, oxidation, and degradation under thermal cycling. Maintaining long-term operational stability under cyclic magnetic fields remains a critical challenge. Embedding nanoparticles into functional matrices (e.g. elastomers, porous scaffolds, or composite heat exchangers) without compromising magnetocaloric or thermal performance is complex. Encapsulation or binder materials may insulate thermally or magnetically, reducing system efficiency. Scalable synthesis techniques such as sol-gel, co-precipitation, or hydrothermal methods often struggle to maintain particle quality, size uniformity, and crystallinity, which are essential for consistent magnetic behavior. Poor crystallinity and wide size distributions lead to variable MCE responses. Moreover, many oxide and intermetallic nanoparticles (e.g. Gd-, LaFeSi-, and Mn-based alloys) are highly sensitive to stoichiometry and surface oxidation. This can alter magnetic properties and degrade MCE performance. Protective coatings like SiO_2_ or polymers are often necessary but may introduce thermal or magnetic barriers [23]. Although particle bed configurations offer a large interfacial area for heat exchange, they are associated with a non-negligible pressure drop [28,29]. Ultimately, reliable integration of magnetocaloric nanoparticles into practical solid-state or fluidic refrigeration systems remains a significant technological bottleneck.

The application of magnetocaloric thin films in magnetic refrigeration, particularly for on-chip cooling, micro-refrigerators, and cryogenic devices, offers exciting opportunities but also presents considerable challenges stemming from dimensional constraints, interfacial effects, and material integration issues. Similar to ferromagnetic nanoparticles, ferromagnetic thin films typically exhibit reduced *T*_*C*_ and Δ*S*_*M*_ compared to their bulk counterparts. These reductions arise from finite-size effects, epitaxial strain, and surface/interface interactions. While *RC* can be enhanced due to broadening of the transition, the strong suppression of Δ*S*_*M*_ limits their effectiveness for active cooling. Epitaxial strain from lattice-mismatched substrates can distort crystal symmetry, suppress magnetic ordering, or shift transition temperatures. In some systems, such strain can enhance Δ*S*_*M*_ through coupling with substrate structural transitions, but the effect is typically confined to a narrow temperature window, thereby reducing *RC*. Interestingly, in weakly antiferromagnetic or mixed-phase (FM + AFM) thin films, the combined influence of strain and reduced dimensionality can enhance the MCE, enabling large Δ*S*_*M*_ values under lower critical magnetic fields. However, thin films inherently possess low thermal mass and limited thermal conductivity, especially in multilayered or oxide-based systems, posing serious challenges for efficient heat extraction and limiting practical cooling capacity. Compared to bulk materials, thin films typically exhibit reduced electrical conductivity due to increased electron scattering at surfaces and interfaces, which leads to lower carrier mobility. Moreover, thin films undergoing FOMTs are prone to performance degradation over repeated thermal or magnetic cycling. For instance, Gd₅Si₂Ge₂ thin films have demonstrated significant Δ*S*_*M*_ loss after ~ 1000 cycles. From a fabrication standpoint, achieving high film quality is challenging due to variations introduced by deposition techniques (e.g. pulsed laser deposition, sputtering). Issues such as grain boundaries, off-stoichiometry, crystalline defects, and oxygen vacancies, especially in complex oxides, can cause inconsistent MCE performance across samples and devices. Only a limited set of magnetocaloric materials (e.g. Gd, Heusler alloys, and certain manganites) have been successfully deposited as high-quality thin films. Maintaining stoichiometry, crystallinity, and magnetic order during deposition remains particularly difficult for multicomponent or intermetallic systems. Furthermore, the thin geometry (typically 10–500 nm) inherently limits volumetric entropy change and cooling power. Scaling up to practical applications necessitates multilayer stacking or large-area film integration, which introduces additional thermal and magnetic engineering complexities. Integrating the MCE with other phenomena, such as the thermoelectric effect, in thin-film systems may offer a promising route to enhance overall cooling efficiency [282], though further investigation is required to validate this approach

Magnetocaloric ribbons, typically fabricated via rapid solidification techniques such as melt spinning, offer advantages like flexibility, high surface area, and fast thermal response, making them promising for use in magnetic refrigeration systems. However, their practical application faces several notable challenges. Compared to their bulk counterparts, ribbons often exhibit lower Δ*S*_*M*_, primarily due to microstructural disorder from rapid solidification, grain texture effects, and diminished long-range magnetic ordering. Many magnetocaloric ribbons, particularly those based on intermetallic compounds such as La(Fe,Si)_13_ and Mn-based Heusler alloys, are mechanically brittle, a result of their crystalline or partially amorphous nature. This brittleness limits their durability under mechanical stress, thermal cycling, or during device integration. Additionally, ribbons generally possess low thermal conductivity, particularly in amorphous or disordered phases, which restricts efficient heat exchange and slows cooling response. Compared to their bulk counterparts, electrical conductivity tends to decrease in magnetic ribbons. The reduction mainly arises from structural disorder (amorphous or nanocrystalline phases), increased grain boundary scattering, and possible surface oxidation. However, the exact difference depends on composition, thickness, and post-processing treatments (e.g. annealing to induce crystallization). For thinner ribbons (typically <50 μm), the small volume further limits the overall cooling capacity, making it difficult to scale up for higher-power applications. Some ribbons undergo FOMT, often accompanied by thermal and magnetic hysteresis, which reduces energy efficiency during cyclic operation and may impact device lifespan under repeated use. Their high surface area also makes them vulnerable to oxidation, especially in ambient or humid environments, degrading their magnetocaloric performance over time. From a fabrication standpoint, achieving compositional and structural uniformity during rapid solidification is challenging, with minor processing variations leading to significant changes in performance. Finally, despite their mechanical flexibility, practical integration of ribbons into magnetic cooling modules (e.g. regenerators or heat exchangers) requires precise alignment and mechanical support while ensuring effective thermal and magnetic coupling – an engineering challenge that remains unresolved.

Magnetocaloric microwires, with cylindrical and flexible geometries ranging from a few micrometers to sub-micron diameters, offer several advantages for magnetic refrigeration applications, including high surface-to-volume ratio, mechanical flexibility, and rapid heat exchange. Despite these benefits, significant challenges hinder their practical implementation. One major issue is the controlled fabrication of high-quality microwires with consistent diameter, uniform composition, and crystallinity. Only a narrow range of magnetocaloric materials can be processed into microwires using methods such as melt extraction, in-rotating-water quenching, or glass-coated melt spinning. Many promising magnetocaloric alloys like La(Fe,Si)_13_ are brittle or chemically unstable during wire processing, limiting material options. Microwires, particularly those composed of intermetallic compounds, tend to be mechanically fragile, especially under repeated thermal or magnetic cycling. Over time, this can lead to microcracks or delamination from protective coatings or composite matrices, compromising structural integrity and performance. In magnetic microwires, the electron mean free path can approach the wire diameter, resulting in enhanced surface scattering. This increased scattering reduces carrier mobility and, consequently, lowers electrical conductivity compared to bulk materials. In practical cooling devices, bundling or aligning large numbers of microwires is required to achieve significant cooling power. However, ensuring uniform magnetic field exposure and efficient thermal contact with the working fluid (liquid or gas) across such arrays is technically demanding. The design of mechanical supports that maintain wire alignment without introducing thermal resistance remains an open engineering challenge. Thermal management is further complicated by poor thermal coupling between wires in dense bundles and between wires and their embedding matrices. Oxidation-preventing coatings may inadvertently act as thermal insulators, impeding heat exchange. Achieving uniform temperature distribution during heating and cooling cycles in densely packed microwire arrays is also difficult, reducing system efficiency. These combined challenges, ranging from fabrication constraints to integration and thermal engineering, must be addressed before magnetocaloric microwires can be effectively utilized in compact, scalable magnetic refrigeration systems. Table 6 summarizes the main advantages and key challenges in the development of low-dimensional magnetocaloric materials.

**Table 6.** The main advantages and key challenges in low-dimensional magnetocaloric materials.

Form	Advantages	Key Challenges	Material & Engineering Constraints
Nanoparticles	–Suitable for cryogenic & localized cooling –Entropy broadening can improve refrigerant capacity	–Reduced Curie Temperature (*T*_*C*_) and Δ*S*_*M*_ –Require high magnetic fields for AFM types (3–7 T) –Low thermal conductivity & high interfacial resistance –Agglomeration, oxidation, degradation under cycling	–Complex integration into matrices –Thermal/magnetic insulation from binders or coatings –Difficult scalable synthesis with consistent quality –Sensitive to stoichiometry, oxidation –Need protective coatings (e.g. SiO₂) that may reduce performance
Thin Films	–On-chip & microcooling potential –Integration with other effects (e.g. thermoelectric) –Potential for large Δ*S*_*M*_ in strained AFM/FM systems	–Reduced *T*_*C*_ and Δ*S*_*M*_ due to finite–size effects, strain –Low thermal mass and conductivity –Δ*S*_*M*_ degradation with cycling (e.g. Gd₅Si₂Ge₂) –Hysteresis losses in FOMT materials	–Deposition–related defects (grain boundaries, off–stoichiometry) –Limited materials with high film quality –Difficulty maintaining magnetic order during deposition –Volume constraints limit cooling power –Multilayer stacking adds complexity
Ribbons	–High surface area –Fast thermal response –Flexible (to some extent)	–Reduced Δ*S*_*M*_ due to disorder and texture –Mechanical brittleness–Low thermal conductivity–Small volume limits cooling capacity–Hysteresis losses in FOMT materials	–Prone to oxidation –Structural/compositional uniformity hard to control –Engineering integration (alignment, thermal coupling) is complex
Microwires	–High surface–to–volume ratio –Mechanical flexibility –Fast heat exchange	–Fragility under thermal/magnetic cycling –Limited materials can be processed as microwires –Oxidation/degradation over time	–Challenging to fabricate uniform wires–Need dense bundles for sufficient cooling–Poor thermal coupling in bundles/matrices– Alignment and support design remains unresolved

## 6. Concluding remarks

Magnetocaloric materials with reduced dimensionality, such as nanoparticles, thin films, ribbons, and microwires, offer promising avenues for the development of energy-efficient magnetic refrigeration technologies. Compared to their bulk counterparts, ferromagnetic nanoparticles and thin films often exhibit lower Curie temperatures and reduced magnetic entropy change, though they may show enhanced refrigerant capacity due to broadened magnetic transitions. In contrast, magnetocaloric ribbons and microwires, typically produced via rapid solidification, can exhibit both enhanced magnetocaloric performance and improved mechanical properties relative to their bulk glassy forms. Particularly notable are antiferromagnetically weakened or mixed-phase FM/AFM nanoparticles and thin films, which outperform bulk antiferromagnets due to their negligible magnetic and thermal hysteresis losses and enhanced MCE performance. These effects are tunable via substrate-induced strain, finite size effects, and surface/interface modifications. In amorphous ribbons and microwires, thermal treatments such as annealing can significantly improve magnetocaloric properties by inducing nanocrystalline phases. However, this often comes at the cost of mechanical fragility, creating a trade-off between magnetocaloric performance and structural durability. Optimizing nanocrystallization conditions may simultaneously enhance both thermal and mechanical properties. This is especially crucial in Heusler alloy-based ribbons and microwires, where precise thermal processing governs performance outcomes.

From a fabrication and scalability standpoint, methods for producing ribbons and microwires are more readily adaptable to practical cooling device architectures, whereas the synthesis of high-quality nanoparticles and thin films remains largely limited to laboratory-scale methods. This underscores the need for new scalable fabrication techniques for reduced-dimensionality materials.

While much of the current research emphasizes achieving high Δ*S*_*M*_ and *RC*, fewer studies have rigorously addressed the adiabatic temperature change (Δ*T*_*ad*_) – a key parameter for evaluating real-world cooling performance. Δ*T*_*ad*_ is technically challenging to measure, particularly in nanoparticle and thin film systems. In some cases, ribbons and microwires exhibit significantly reduced Δ*T*_*ad*_ compared to their bulk analogs, necessitating further comprehensive investigations into this metric for all reduced-dimensionality formats.

Theoretically, magnetocaloric materials with enhanced surface areas (e.g. nanoparticles, ribbons, microwires) are predicted to offer superior heat exchange with the surrounding environment, boosting cooling efficiency. However, these models often overlook the influence of structural assembly materials, such as binders, matrices, and coatings, on overall system performance. In practice, interactions between particles or wires, as well as between layers in laminated structures, can significantly modify the magnetic and thermal behavior. These interfacial and collective effects require thorough theoretical and experimental scrutiny when engineering composite or structured cooling devices.

In summary, while magnetocaloric materials with reduced dimensionality hold great promise, numerous technical barriers, ranging from synthesis and processing to integration and thermal management, must be systematically addressed. Only through coordinated advances in materials science, device engineering, and system-level optimization can these materials be effectively utilized in compact, scalable, and high-performance magnetic refrigeration systems.
